# Maltreatment predicts centiles of individual variation from normative brain growth in children

**DOI:** 10.1038/s42003-026-11143-6

**Published:** 2026-10-22

**Authors:** Johannes Julius Mohn, Martin Bauer, Ferdinand Hoffmann, Jerod Rasmussen, Anna Tietze, Sonja Entringer, Sibylle M. Winter, Claudia Buss, Christine Heim

**Affiliations:** 1 Institute of Medical Psychology, Charité – Universitätsmedizin Berlin, corporate member of Freie Universität Berlin, Humboldt-Universität zu Berlin, Berlin, Germany; 2 Max Planck School of Cognition, Max Planck Institute for Human Cognitive and Brain Sciences, Leipzig, Germany; 3 Experimental and Clinical Research Center, Charité – Universitätsmedizin Berlin, corporate member of Freie Universität Berlin, Humboldt-Universität zu Berlin, Berlin, Germany; 4 German Center for Mental Health (DZPG), Berlin-Potsdam Partner Site, Berlin, Germany; 5 Development, Health and Disease Research Program, School of Medicine, University of California, Irvine, CA USA; 6 Department of Pediatrics, University of California, Irvine, CA USA; 7 Institute of Neuroradiology, Charité – Universitätsmedizin Berlin, corporate member of Freie Universität Berlin, Humboldt-Universität zu Berlin, Berlin, Germany; 8 Department of Child and Adolescent Psychiatry, Charité – Universitätsmedizin Berlin, corporate member of Freie Universität Berlin, Humboldt-Universität zu Berlin, Berlin, Germany; 9 German Center for Child and Adolescent Health (DZKJ), Berlin Partner Site, Charité – Universitätsmedizin Berlin, Berlin, Germany; 10 NeuroCure Cluster of Excellence, Charité – Universitätsmedizin Berlin, corporate member of Freie Universität Berlin, Humboldt-Universität zu Berlin, Berlin, Germany

**Keywords:** Risk factors, Neuroscience

## Abstract

Exposure to maltreatment in childhood is posited to profoundly disrupt brain development with adverse consequences for cognitive development and health across the lifespan. Here we use “brain charts” from the largest available neuroimaging database to quantify individual brain volumes relative to normative brain growth distributions in 299 children, aged 3 to 13 years, including 123 children with clinically verified maltreatment exposure. Using magnetic resonance imaging, we obtain volumes of cortical grey, total white, and subcortical grey matter and derive chart-based centiles for each child and tissue type. In our sample, children with maltreatment exposure exhibit lower mean brain centiles across tissues than unexposed children. Associations of greater maltreatment severity with lower brain centiles do not survive correction for multiple comparisons. Lower brain centiles are associated with lower intellectual ability and more behavioural problems. These findings provide evidence for widespread differences in brain structure associated with childhood maltreatment and their relevance to intellectual ability and behavioural functioning.

## Introduction

Neuroplasticity is essential for learning and cognitive development^1^, but it may also render the developing brain vulnerable to adverse experiences such as childhood maltreatment^2^. Human and animal studies suggest that chronic stress and elevated glucocorticoid levels exert neurotoxic effects^3,4^, particularly during sensitive periods^5–7^. These disruptions to neuroplastic processes may profoundly alter trajectories of brain development and behaviour, with lasting consequences for intellectual ability^8,9^ and elevated psychiatric risk^10,11^.

Neuroimaging studies have consistently linked childhood maltreatment and neurodevelopmental outcomes^12–14^. Severe institutional deprivation in early childhood has been associated with reductions in brain volume and cognitive deficits in adulthood despite subsequent placement in foster families^15^. Similarly, in a small sample of young children imaged shortly after maltreatment exposure, greater maltreatment severity correlated with smaller brain size, which in turn was associated with lower intelligence^16^. The generalizability of these findings is, however, limited due to the lack of a control group, which underscores the need for comparisons against developmental benchmarks. Longitudinal studies further demonstrate that earlier removal from adverse caregiving environments predicts more typical cortical development during adolescence, suggesting that psychosocial neglect influences developmental trajectories rather than only cross-sectional brain differences^17^. More recently, normative modelling has extended this work by demonstrating that broad categories of adverse experiences are associated with deviations from typical developmental trajectories in adulthood^18^, whereas a recent large-scale study found no significant associations between maltreatment and normative brain measures in its adolescent age group^19^. However, whether maltreatment-related deviations from developmental benchmarks are already detectable during childhood—a period of rapid brain growth and heightened neuroplasticity—remains unclear and requires neuroimaging studies in children with verified maltreatment exposure.

Recently, brain growth charts, derived from over 120,000 brain scans, have become available and provide a novel quantitative framework for situating individual brain volumes relative to age- and sex-matched norms^20^. Analogous to paediatric growth charts for height and weight, they allow researchers and clinicians to determine whether a child’s brain volume falls within the expected range for their developmental stage or deviates substantially from it, providing an intuitive and standardized measure of neurodevelopmental status^21^. Brain centiles demonstrate longitudinal stability within subjects and robustness to variations in data acquisition across studies^20^. As a result, centiles provide a standardized and directly clinically interpretable metric of individual variation in brain structure. Importantly, brain charts extend cross-sectional case-control analyses by placing group differences within a shared developmental reference that offers insight into the magnitude, direction, and heterogeneity of deviations from typical development^22^.

Leveraging the availability of brain charts, we investigated associations between maltreatment exposure and centiles of global brain structure in children aged 3 to 13 years. We recruited children with documented maltreatment exposure verified through structured clinical interviews and children without significant early adversity. This clinically well-characterized cohort addresses an important limitation of the existing literature, in which maltreatment has frequently been assessed retrospectively in adults, while neuroimaging studies of children with clinically verified exposure remain scarce^2^. Using high-resolution structural magnetic resonance imaging (MRI), we derived individualized centiles for cortical grey matter, total white matter, and subcortical grey matter by charting brain volumes against the largest population reference of brain growth to date^20^. We then investigated deviations from the population mean and differences in brain centiles between children with and without childhood maltreatment exposure within the sample. This complementary comparison provides a more stringently characterized reference group because maltreatment exposure was not systematically assessed in the reference datasets underlying the normative brain charts, meaning that the normative population is likely to include a non-negligible proportion of children with maltreatment histories. We further examined associations of brain centiles with maltreatment severity, intellectual ability and behavioural problems. We observed brain volumes significantly lower than expected for their age and sex in children with maltreatment exposure. Associations of greater maltreatment severity with lower brain centiles did not survive correction for multiple comparisons. Moreover, lower brain centiles were associated with lower intellectual ability and more internalizing and externalizing behaviours. Thus, we provide evidence for maltreatment-related deviations from normative brain structure distributions during childhood, with consequences for intellectual ability and behaviour.

## Results

### Sample characteristics

This study included 299 children aged 3 to 13 years (48.5% females; mean age = 8.20 ± 1.97 years). Of these, 123 (41.1%) had maltreatment exposure (CM+), verified using the Maternal Maltreatment Classification Interview and coded according to the Maltreatment Classification System^23^, and 176 were children without exposure to maltreatment (CM−). Demographic characteristics of the sample are summarized in Table 1.

**Table 1:** Demographic variables and group differences

	Total (*n* = 299)	CM− (*n* = 176)	CM+ (*n* = 123)	*p*
Age (years) Mean (SD) Median [Min, Max]	8.20 (1.97) 8.25 [3.34, 13.1]	8.04 (1.92) 8.20 [3.39, 12.2]	8.44 (2.04) 8.59 [3.34, 13.1]	0.088
Sex Male Female	145 (48.5%) 154 (51.5%)	81 (46.0%) 95 (54.0%)	64 (52.0%) 59 (48.0%)	0.365
SES (composite) Mean (SD) Median [Min, Max] Missing	15 (5.44) 16 [3, 21] 4	17.8 [3.36] 19 [6, 21] 0	10.9 (5.31) 11 [3, 21] 4	0.0000000000000002

Values are shown as mean (SD) for continuous variables and *n* (%) for categorical variables. *P* values refer to Welch two-sample *t*-tests for age and SES and chi-square test for sex. All statistical tests were two-sided unless otherwise specified.

*CM+* childhood maltreatment exposure, *CM−* non-exposed, *SES* socioeconomic status.

### Childhood maltreatment and brain centiles

Linear mixed-effects models first tested whether brain centiles differed from age- and sex-normed population means and subsequently compared brain centiles between maltreatment groups, adjusting for residual effects of age and sex and accounting for non-independence among siblings using a family-level random effect. Children in the CM+ group ranked around the 40th percentile for cortical grey matter volume (cGM, 38th percentile, 95% CI = [32.63, 43.32], *p*_FDR_ < 0.001), total white matter volume (WM, 42nd percentile, 95% CI = [36.93, 46.12], *p*_FDR_ = 0.001) and subcortical grey matter volume (sGM, 39th percentile, 95% CI = [34.22, 42.83], *p*_FDR_ < 0.001).

**Fig. 1:**
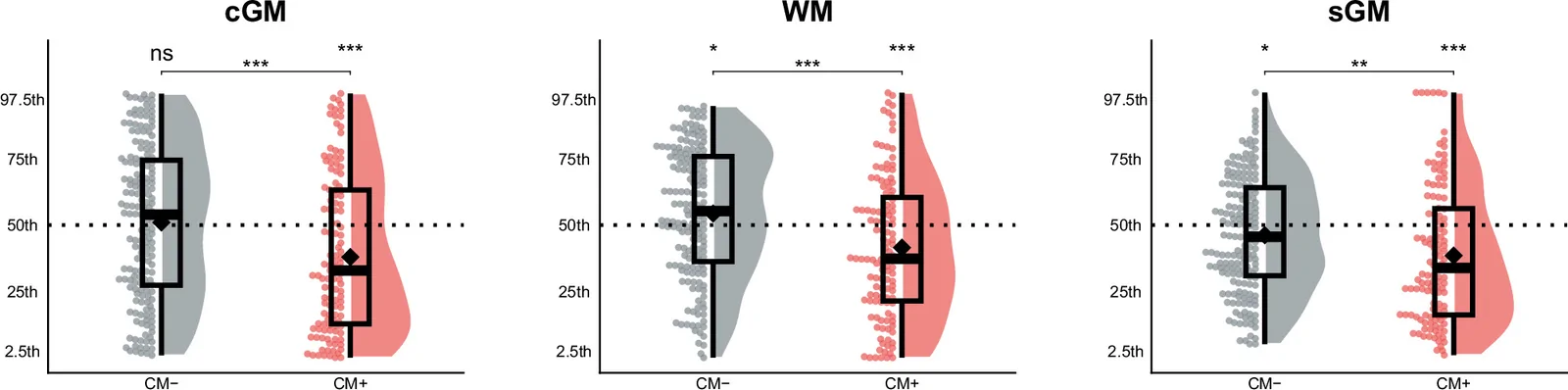
Maltreatment-related differences in global brain centiles. Estimated marginal mean contrasts between the CM+ (*n* = 123) and CM− (*n* = 176) groups and differences from population means. Individual data points represent individual children. The dotted line represents the population mean (50th percentile). Asterisks above the group distributions indicate differences from the population mean; asterisks with brackets compare groups and denote statistical significance: * *p* < 0.05, ** *p* < 0.01, *** *p* < 0.001. **cGM**, cortical grey matter: CM+ vs. population mean, *p* = 0.0000278; CM− vs. population mean, *p* = 0.647; CM+ vs. CM−, *p* = 0.00420. **WM**, total white matter: CM+ vs. population mean, *p* = 0.000676; CM− vs. population mean, *p* = 0.0248; CM+ vs. CM − , *p* = 0.0000998. **sGM**, subcortical grey matter: CM+ vs. population mean, *p* = 0.000000636; CM− vs. population mean, *p* = 0.0405; CM+ vs. CM−, *p* = 0.00730. *P* values refer to linear mixed-effects models adjusted for age and sex with a random intercept for family and were adjusted using the false discovery rate. Box plot elements: The centre line represents the median; box limits denote the upper and lower quartiles (Q3 and Q1), spanning the interquartile range (IQR); whiskers extend to the smallest and largest values within 1.5 × IQR beyond the quartiles. Diamonds denote group means.

Brain centiles were also significantly lower in the CM+ than in the CM− group for cGM (adjusted mean difference = −13.07, 95% CI [−20.06, −6.08], *p*_FDR_ < 0.001), WM (−12.88, 95% CI [−18.89, −6.88], *p*_FDR_ < 0.001), and sGM (−7.72, 95% CI [−13.34, −2.10], *p*_FDR_ = 0.007). Together, these findings indicate that children exposed to maltreatment had significantly smaller brain volumes than expected for their age and sex as well as smaller brain volumes relative to unexposed children (Fig. 1).

### Maltreatment severity and brain centiles

**Fig. 2:**
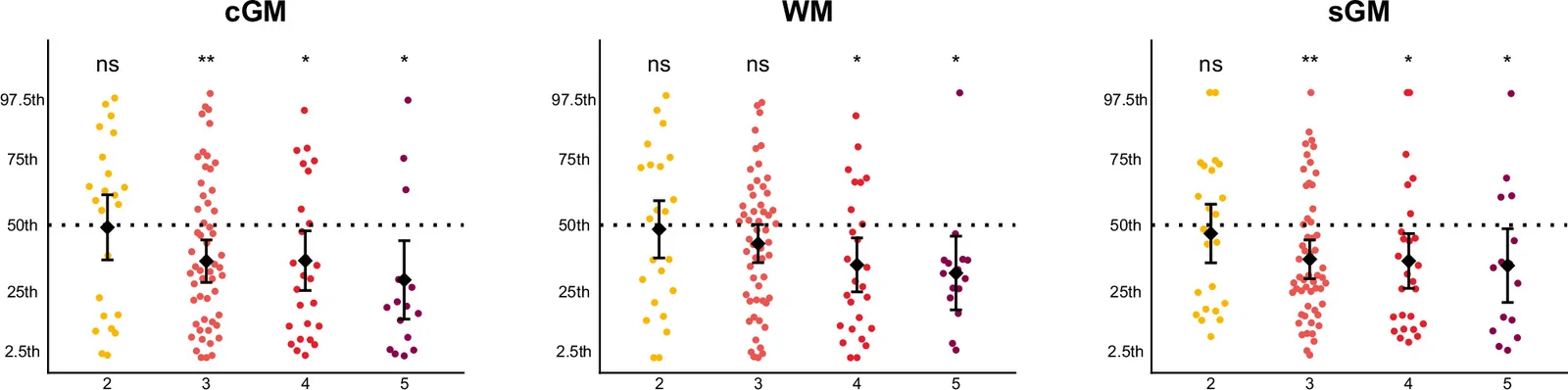
Associations of maltreatment severity with brain centiles. Individual data points represent individual children. Colour gradient from yellow to dark red represents increasing maltreatment severity (severity 2, *n* = 23; severity 3, *n* = 55; severity 4, *n* = 26; severity 5, *n* = 15). Black diamonds and error bars represent mean centiles and 95% confidence intervals. Dotted line represents the population mean (50th percentile). Asterisks above the point clouds denote statistical significance from the population mean: * *p* < 0.05, ** *p* < 0.01, *** *p* < 0.001. **cGM**, cortical grey matter: severity 2, *p* = 0.890; severity 3, *p* = 0.00427, severity 4, *p* = 0.0266, severity 5, *p* = 0.0134. **WM**, total white matter: severity 2, *p* = 0.776; severity 3, *p* = 0.0746, severity 4, *p* = 0.0169, severity 5, *p* = 0.0226. **sGM**, subcortical grey matter: severity 2, *p* = 0.573; severity 3, *p* = 0.00283, severity 4, *p* = 0.0215, severity 5, *p* = 0.0423. All *p* values were adjusted for multiple comparisons using the false discovery rate.

To characterize the relationship between maltreatment severity and brain centiles, we defined maltreatment severity as the highest maltreatment rating across all maltreatment types a child had experienced. Maltreatment severity, modelled as a continuous predictor, was not significantly associated with cGM (*β* = −0.06, 95% CI [−0.11, −0.00], *t*(112.63) = − 2.04, *p* = 0.044, *p*_FDR_ = 0.066), WM (*β* = −0.06, 95% CI [−0.11, −0.01], *t*(115.00) = − 2.32, *p* = 0.022, *p*_FDR_ = 0.066), or sGM (*β* = −0.04, 95% CI [−0.08, 0.02], *t*(114.99) = −1.38, *p* = 0.172, *p*_FDR_ = 0.172) after controlling for multiple comparisons (Supplementary Table S1), but children who experienced maltreatment with a severity rating of 3 or higher had on average significantly lower brain centiles than the population norm (Fig. 2).

### Childhood maltreatment and intellectual ability

Intellectual ability was assessed using the Snijders-Oomen Nonverbal Intelligence Test (SON-R)^24^, yielding standardized intelligence quotients (IQ), and the Verbal Comprehension Scale from the Wechsler Intelligence Scale for Children (WISC)^25^. Children with maltreatment exposure had lower IQ and verbal comprehension scores compared to non-exposed children (SON-R, adjusted mean difference = −13.59, 95% CI [−17.52, −9.66], *t*(258.84) = −6.80, *p*_FDR_ < 0.001; WISC, −14.90, 95% CI [−19.04, −10.77], *t*(254.78) = − 7.10, *p*_FDR_ < 0.001; Table 2, Supplementary Fig. S1). Greater maltreatment severity was associated with lower scores (SON-R, *β* = −6.33, 95% CI [ − 9.39, −3.27], *t*(111.11) = −4.10, *p*_FDR_ < 0.001; WISC, *β* = −4.58, 95% CI [−7.47, −1.70], *t*(86.71) = −3.16, *p*_FDR_ = 0.002).

**Table 2:** Intellectual ability and psychopathological symptoms

	Total (*n* = 299)	CM− (*n* = 176)	CM+ (*n* = 123)	*p*
Nonverbal IQ (SON-R) Mean (SD) Median [Min, Max] Missing	104 (17.4) 103 [55, 145] 6	109 (15.8) 109 [64, 145] 2	95.8 (16.8) 95 [55, 145] 4	0.0000000000712
Verbal IQ (WISC) Mean (SD) Median [Min, Max] Missing	106 (18.3) 106 [40, 155] 6	112 (3.20) 113 [40, 155] 3	97.1 (4.49) 95 [59, 139] 3	0.0000000000254
Internalizing (SDQ) Mean (SD) Median [Min, Max] Missing	3.76 (3.49) 3.00 [0, 17] 18	2.59 (2.71) 2.00 [0, 11] 1	5.69 (3.79) 5.00 [0, 17] 17	0.000000000000000192
Externalizing (SDQ) Mean (SD) Median [Min, Max] Missing	5.84 (4.22) 5.00 [0, 19] 18	4.30 (3.20) 4.00 [0, 14] 1	8.39 (4.49) 9.00 [0, 19] 17	0.0000000000103

Values are shown as mean (SD) and median [min, max]. *P* values refer to linear mixed-effects models controlling for age and sex with a random intercept for family and were adjusted for multiple comparisons using the false discovery rate.

*SON-R* Snijders-Oomen Non-verbal Intelligence Test, *WISC* Wechsler Intelligence Scale for Children, Verbal Comprehension Scale, *SDQ* Strengths and Difficulties Questionnaire.

### Childhood maltreatment and behavioural problems

Children with maltreatment exposure scored higher than non-exposed children on externalizing (adjusted mean difference = 4.16, 95% CI [3.24, 5.07], *t*(231.31) = 8.98, *p*_FDR_ < 0.001) and internalizing behaviours (2.93, 95% CI [2.12, 3.74], *t*(244.42) = 7.15, *p*_FDR_ < 0.001) assessed with the Strengths and Difficulties Questionnaire (SDQ)^26^ (Table 2, Supplementary Fig. S1). Moreover, externalizing and internalizing behaviours were positively associated with maltreatment severity (externalizing, *β* = 1.25, 95% CI [0.32, 2.18], *t*(100.00) = 2.66, *p*_FDR_ = 0.018; internalizing, *β* = 0.78, 95% CI [0.03, 1.53], *t*(88.03) = 2.06, *p*_FDR_ = 0.042).

### Brain centiles, intellectual ability, and behavioural problems

**Fig. 3:**
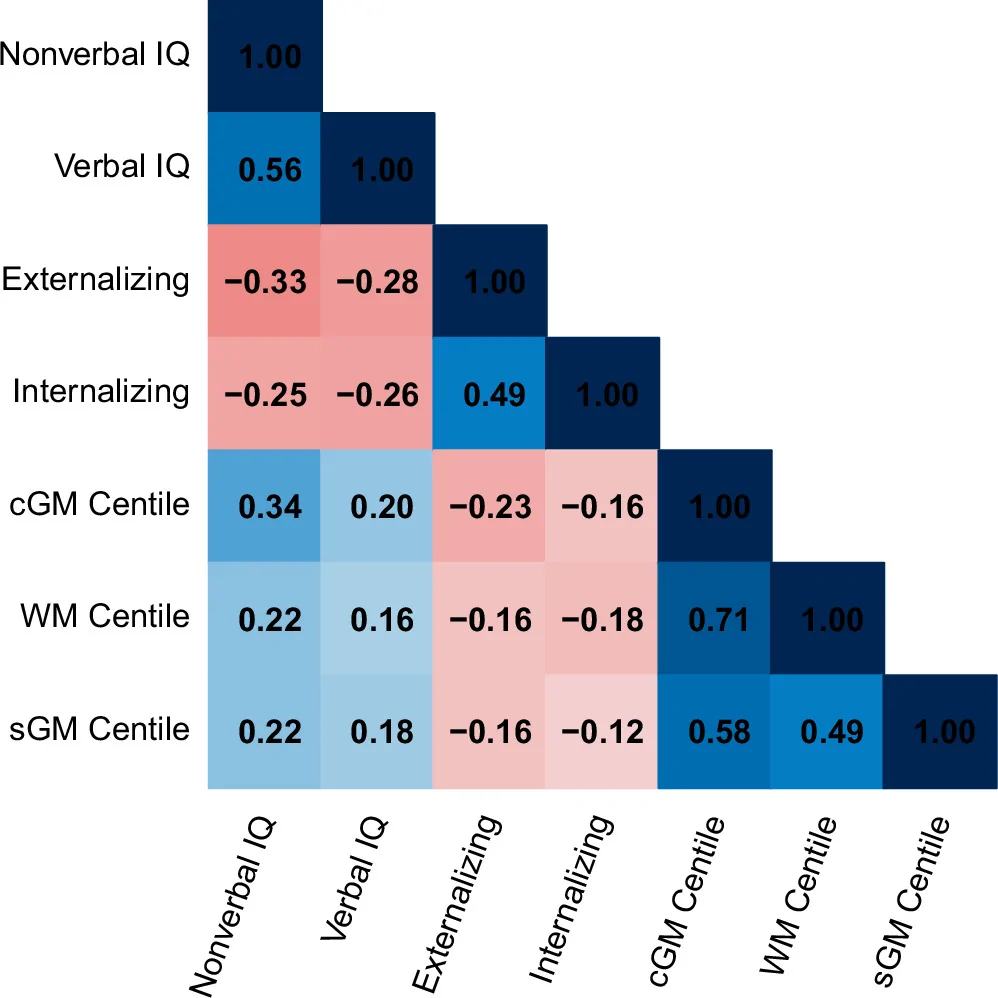
Bivariate associations among brain, cognitive, and behavioural measures. Bivariate Pearson correlations among brain centiles, nonverbal IQ (SON-R), verbal IQ (WISC, Verbal Comprehension Scale), and externalizing and internalizing scores (SDQ). Values indicate Pearson correlation coefficients (*r*). Colour indicates the direction and magnitude of the correlation coefficient, ranging from deep red for stronger negative correlations to deep blue for stronger positive correlations, with lighter colours indicating correlations closer to zero. cGM, cortical grey matter; WM, total white matter; sGM, subcortical grey matter.

Brain centiles were associated with intellectual ability and behavioural problems. Higher cGM, WM and sGM centiles were associated with higher nonverbal IQ (SON-R; *β* = 14.71–19.95, all *p*_FDR_ ≤ 0.001) and verbal comprehension (WISC; *β* = 10.55–12.13, all *p*_FDR_ ≤ 0.014). Higher brain centiles were also associated with fewer externalizing (*β* = −3.13 to −2.58, all *p*_FDR_ ≤ 0.013) and internalizing problems (*β* = −2.37 to −2.04, all *p*_FDR_ ≤ 0.013; Fig. 3, Table 3 and Supplementary Fig. S3). There was no evidence that these associations differed by childhood maltreatment exposure (Supplementary Table S3).

**Table 3:** Associations among brain centiles, intellectual ability, and behavioural problems

Outcome	Imaging Phenotype	Estimate	95% CI	*t*	df	*p*	Adjusted *p*
SON-R	cGM	19.95	13.48, 26.43	6.06	287.47	0.00000000418 ***	0.0000000501 ***
WISC	cGM	12.13	5.07, 19.19	3.38	288.96	0.000821 ***	0.00169 **
SON-R	WM	15.94	8.45, 23.43	4.19	283.54	0.0000371 ***	0.000223 ***
WISC	WM	11.79	3.95, 19.63	2.96	256.76	0.00335 **	0.00503 **
SON-R	sGM	14.71	6.64, 22.78	3.59	274.81	0.000393 ***	0.00118 **
WISC	sGM	10.55	2.16, 18.93	2.48	247.97	0.0139 *	0.0139 *
Externalizing	cGM	−3.13	−4.78, −1.49	−3.76	263.66	0.000208 ***	0.000834 ***
Internalizing	cGM	−2.27	−3.60, −0.95	−3.38	264.68	0.000844 ***	0.00168 **
Externalizing	WM	−2.58	−4.51, −0.64	−2.62	270.90	0.00923 **	0.0123 *
Internalizing	WM	−2.37	−3.86, −0.87	−3.12	218.12	0.00202 **	0.00347 **
Externalizing	sGM	−2.69	−4.77, −0.60	−2.53	276.97	0.0118 *	0.129 *
Internalizing	sGM	−2.04	−3.60, −0.48	−2.58	179.31	0.0106 *	0.0127 *

Estimates and 95% confidence intervals are beta coefficients from linear mixed-effects models adjusted for age and sex with a random intercept for family. *P* values were adjusted for multiple comparisons using the false discovery rate. Asterisks denote statistical significance: * *p* < 0.05, ** *p* < 0.01, *** *p* < 0.001.

### Moderation analyses

We tested whether socioeconomic status (SES), a well-established predictor of intellectual and brain development in children, moderated the associations between maltreatment exposure and brain centiles by including an interaction term of maltreatment group status and SES in the main linear mixed-effects models while controlling for age, sex, and family clustering. SES was derived from parental education, occupation, and monthly net income^27^ and differed between groups, with higher SES in the CM− group (Table 1). SES moderated the linear association between childhood maltreatment and cGM centiles, but this result did not survive correction for multiple comparisons (*β* = −0.09, 95% CI [ − 0.18, −0.00], *t*(269.49) = − 2.00, *p* = 0.046, *p*_FDR_ = 0.138). There was no evidence for SES moderation of associations with WM (*β* = −0.02, 95% CI [−0.10, 0.06], *t*(274.02) = − 0.46, *p* = 0.643, *p*_FDR_ = 0.643) and sGM (*β* = −0.04, 95% CI [−0.12, 0.03], *t*(271.42) = − 1.21, *p* = 0.226, *p*_FDR_ = 0.339; Supplementary Table S2, Fig. S2).

## Discussion

In this study, we applied normative brain growth charts to express structural MRI data as centiles^20^ and investigate differences in brain structure associated with childhood maltreatment. Children aged 3 to 13 years with clinically verified histories of childhood maltreatment showed lower cortical grey matter, total white matter, and subcortical grey matter volumes than expected for their age and sex. By leveraging large-scale normative models in a well-characterized sample of children, our findings provide evidence for differences in global brain structure associated with maltreatment. Moreover, lower brain centiles were associated with lower intellectual ability and increased behavioural problems, highlighting the functional relevance of the observed differences. Together, our findings emphasize the pressing need for future studies elucidating how maltreatment shapes early neurodevelopment at neurobiological and molecular levels and informing the development of mechanism-driven interventions.

The present findings extend a growing body of evidence linking childhood maltreatment to differences in brain structure^5,12,14^ by observing deviations of brain volumes from normative developmental distributions. Children with confirmed maltreatment exposure ranked lower across cortical grey matter, subcortical grey matter, and white matter centiles, which is consistent with previous studies reporting smaller global and regional brain volumes associated with childhood maltreatment^15,16,18,19,28^. Notably, a recent large-scale normative modelling study found no significant associations with brain structure in its child and adolescent age group^19^. Differences in the assessment of maltreatment may contribute to these discrepant findings, as maltreatment in that study was assessed using questionnaire self-report, whereas the present study used detailed clinical assessment of exposure in children recruited through child welfare and protection services. We extend this literature by quantifying deviations relative to normative brain growth charts in children with clinically verified maltreatment. Together, the lower brain centiles observed relative to population norms and our carefully characterized group of unexposed children provide complementary evidence for differences in global brain structure associated with maltreatment.

Normative brain charts complement conventional case-control comparisons by charting each child’s brain volume against an age- and sex-specific reference distribution. Their value in the present study lies in expressing individual differences on an intuitive, population-referenced scale and helping to characterize heterogeneity that may be obscured by group averages. For example, normative modelling in ADHD showed that group-level differences were only weakly reflected at the individual level, challenging the notion of the “average patient”^29^, and similar work in autism suggests that group-level differences can be driven by subgroups of individuals with extreme deviations^30^. In our study, despite lower mean centiles in the CM+ group, children in both groups spanned a broad and substantially overlapping range of the normative distribution. Thus, lower average brain centiles associated with maltreatment do not constitute a uniform neuroanatomical phenotype at the individual level, nor do our findings establish clinical utility of brain centiles for identifying individual children with maltreatment exposure. Establishing clinical utility will require prospective validation of their predictive performance for developmental outcomes in independent samples. The observed variation was not clearly explained by maltreatment severity, for which associations with brain centiles did not survive correction for multiple comparisons. Understanding why children with maltreatment exposure occupy markedly different positions within the normative distribution, and whether factors associated with vulnerability or resilience contribute to this variation, represents important questions for future longitudinal studies.

Previously, we observed differences in total grey but not white matter in younger children and proposed that grey matter may be particularly sensitive to adversity during early childhood^16^. This difference from the present findings may relate to the distinct developmental timing of grey and white matter changes. Human brain development begins in gestation, and distinct mechanisms of neuroplasticity govern this process well into early adulthood. While synaptogenesis predominates during early brain development, with synaptic density reaching its peak between 2 and 7 years of age^31,32^, white matter has a protracted development. Differences in global white matter associated with maltreatment may therefore become more detectable with increasing age, although longitudinal data are required to test this possibility. More nuanced characterization of white matter tracts and myelination requires specialized neuroimaging techniques like diffusion tensor imaging^33^.

The associations of brain centiles with intellectual ability and behavioural problems further highlight the functional relevance of the observed maltreatment-related differences in brain structure. Lower brain centiles were associated with lower IQ scores, consistent with studies documenting maltreatment-related impairments in cognition and intellectual performance^34^ and meta-analytic evidence robustly linking larger brain size to higher IQ^35^. In previous work, we observed persistent negative associations between maltreatment and IQ over a two-year period in preschool children^36^. Lower brain centiles were also associated with higher levels of behavioural problems, which were elevated among children exposed to maltreatment. Together, these findings suggest that individual differences in brain centiles associated with maltreatment are also related to cognitive and behavioural functioning. Longitudinal studies are needed to establish how these associations unfold over development and whether brain structural differences contribute to the emergence or persistence of cognitive and psychiatric difficulties following maltreatment^37,38^.

Drawing largely from animal models that provide causal evidence, and supported by studies in humans, research suggests that adverse experiences during sensitive periods can have lasting effects on brain development^2,3,39^. Although our findings indicate that maltreatment-related experiences are associated with deviations from age- and sex-specific expectations for brain structure, we can only speculate how the observed deviations may arise. One possibility is that when developmentally expected inputs from the environment are absent, as is the case in neglect, typical neurocognitive development is disrupted, which may lead to aberrant brain structure changes^40^. Another possibility is that in the context of adverse experiences early in life, the brain adapts in anticipation of future adversity in an experience-adaptive manner^41^. Similarly, it has been proposed that early life adversity can lead to developmental acceleration, possibly at the cost of reaching a potential peak^42^. It is possible, therefore, that lower than expected brain centiles in children with maltreatment experiences reflect faster synaptogenesis and pruning during early brain development, which presents as overall smaller brain sizes as measured by MRI. Alternatively, the observed deviations in brain structure may reflect consequences of stress^3^. It has been suggested that maltreatment impacts regulatory systems, including stress hormone and immune systems, leading to the increased release of glucocorticoids and inflammatory cytokines, which may in turn signal back into the brain and lead to structural changes^39^. Consistent with the potential relevance of glucocorticoid signalling, experimental work in human induced pluripotent stem cell models has identified transcriptional responses to glucocorticoid exposure involving genes associated with psychopathology^43^. Integrating developmental neuroimaging cohorts with human cell models of early adversity will be essential to understanding how adversity becomes neurobiologically embedded.

Maltreatment rarely occurs in isolation but is embedded within a broader psychosocial context that may shape brain structure. In sensitivity analyses, we therefore examined whether socioeconomic status (SES), a well-established predictor of intellectual and brain development in children, moderated associations between maltreatment exposure and brain centiles. We found no evidence of SES moderation; nevertheless, low SES has been described as an early life adversity in its own right^44^ and frequently co-occurs with maltreatment, as was also evident in our sample. Understanding brain differences associated with maltreatment therefore requires consideration of children’s broader psychosocial environment, which encompasses adverse and protective factors^45^. Multivariate approaches have begun to characterize common and cumulative patterns of adversity^46–49^, although greater integration may come at the cost of specificity regarding individual exposures.

A particular strength of this study is the combination of clinically assessed maltreatment exposure in a large sample of children with child-adapted state-of-the-art MRI acquisition and processing, thus addressing important limitations of a literature that has largely relied on retrospective assessment of childhood maltreatment in adults. Nevertheless, several limitations should be considered. First, the cross-sectional design precludes inferences about how differences in brain structure associated with maltreatment change over time. Whether children with lower brain centiles can move closer to the normative mean over time and which tailored interventions can optimally support this process are questions future longitudinal studies should address^50^. Second, limited sample sizes at higher levels of maltreatment severity may have reduced sensitivity to associations with severity; relatedly, the timing and duration of maltreatment could not be explicitly modelled. Onset and duration tend to be correlated, such that children exposed earlier also tend to be exposed to more episodes of maltreatment as they grow up. Our cohort is characterized by a wide age range, and maltreatment timing is heterogeneous. Larger longitudinal or harmonized samples are needed to disentangle associations with maltreatment timing and duration and to investigate potential sensitive periods^51^. Third, behavioural outcomes were assessed via caregiver report, which may be influenced by reporting biases. Fourth, maltreatment exposure was not systematically assessed in the datasets underlying the normative brain charts, which may therefore include a non-negligible proportion of children with maltreatment histories. This could attenuate the observed differences from the population norm and thus lead to an underestimation of maltreatment-associated differences in brain centiles. Our complementary comparison with our carefully characterized CM− group partly addresses this limitation. In addition, the present study focused on global grey and white matter because current brain growth charts are limited to these global metrics, although normative models for higher-resolution structure and other neuroimaging phenotypes are becoming increasingly available^52–54^. Brain function should also be considered^45^, as strong evidence links childhood maltreatment to altered brain function, including neural responses to stress, threat, reward, and during learning^55–61^, as well as deviations from age-typical changes in resting-state functional connectivity^62^ and the coupling between brain structure and function^63^. Finally, the predominantly European sample may limit generalizability.

To conclude, exposure to childhood maltreatment is a profound and, importantly, preventable risk factor for adverse health outcomes and lower intellectual ability. Consistent with the notion that maltreatment experiences may become biologically embedded in altered neurodevelopment, the current study provides evidence for deviations from normative brain growth across major tissue types in children with clinically verified maltreatment exposure. Lower brain centiles were associated with lower intellectual ability and increased behavioural problems. The substantial individual variation observed among exposed children highlights the importance of future studies to identify factors that confer vulnerability or resilience and may ultimately inform the development of targeted interventions for children who experience maltreatment or are at risk.

## Methods

### Study design and procedure

This study used data from the Kids2Health study, a consortium-led project on the adverse health consequences of childhood maltreatment funded by the German Federal Ministry of Education and Research. Neurocognitive testing was conducted at the Institute of Medical Psychology, Charité – Universitätsmedizin Berlin, and MRI scanning at the Berlin Center for Advanced Neuroimaging (BCAN). The study was approved by the ethics committee of the Faculty of Medicine, Ludwig-Maximilians University Munich under #18-444. All procedures were performed in accordance with the Ethical Principles for Medical Research as established by the World Medical Association Declaration of Helsinki. Parents or legal guardians gave informed written consent and children verbal assent after procedures were fully explained. Participants received monetary compensation for their participation. All families received feedback regarding the child’s health and developmental status and recommendations for follow-up where necessary. Data were stored and managed using Research Electronic Data Capture (REDCap) software^64,65^.

### Participants

A total of 420 children (212 with maltreatment exposure) were recruited, and all were invited to MRI scanning. Of these, 330 completed scans, after which 29 datasets were excluded due to insufficient scan quality and two participants withdrew consent. The final sample comprised 299 children. Children were recruited from the metropolitan area of Berlin, Germany. Children with maltreatment exposure were recruited from child welfare and protection services, including government offices for child welfare, family assistants and counsellors, and agencies of the child welfare sector. Children for the comparison group were recruited via advertisements. Participant’s sex was based on chromosomal sex.

### Maltreatment

Trained clinicians conducted the Maternal Maltreatment Classification Interview with caregivers to corroborate incidents, and maltreatment was coded using the Maltreatment Classification System^23^, which distinguishes physical, sexual, and emotional abuse; failure to provide; lack of supervision; moral-legal maltreatment; and educational maltreatment. Failure to provide and lack of supervision were combined into physical neglect, and moral-legal maltreatment and educational maltreatment were combined into emotional neglect due to conceptual overlap, small numbers of cases, and to facilitate comparability with studies using the Childhood Trauma Questionnaire^66^. Each incident was assigned to one of the five maltreatment subtypes and rated for severity from mild (1) to life-threatening (5). Following prior work^23^, exposure was defined as severity ≥2, except for physical and sexual abuse, for which any verified occurrence was sufficient. Children with at least one exposure were classified as “verified childhood maltreatment” (CM+). Maltreatment severity was calculated as the maximum severity rating across all five subscales.

### Structural neuroimaging acquisition and processing

Structural magnetic resonance images were acquired on a Siemens PRISMAFit 3 T scanner (Siemens, Erlangen, Germany) using a standard 64-channel head coil, with 3D T1-weighted (MPRAGE) and T2-weighted (SPACE) sequences with the following parameters: T1w, TE = 2.22 ms, TR = 2,400 ms, flip angle = 8°, bandwidth = 220 Hz/Px, FOV = 256 mm, resolution 0.8 mm isotropic, TA = 6:38 min; T2w, TE = 563 ms, TR = 3,200 ms, bandwidth = 744 Hz/Px, FOV = 256 mm, resolution 0.8 mm isotropic, TA = 5:57 min. All MRI data were organized according to the BIDS standard^67^. To reduce anxiety and motion, children completed a mock scanner training framed as a “space mission” aboard a “star ship” (MRI scanner) prior to scanning.

MRI data were processed with the ABCD-HCP pipeline optimized for children and youth (freely available at https://github.com/DCAN-Labs/abcd-hcp-pipeline)^68,69^. In brief, the ABCD-HCP anatomical pipeline comprises three stages. First, PreFreesurfer performs brain extraction, denoising, and bias field correction on the T1w and T2w anatomical images and normalizes anatomical data to a standard template. ANTs^70^ DenoiseImage utilizes a Rician distribution model to eliminate scanner noise, and ANTs N4BiasFieldCorrection smooths relative image histograms in different parts of the brain to improve bias field correction. Second, FreeSurfer^71,72^ segments subcortical brain structures, reconstructs cortical surfaces from the normalized anatomical data, and registers them to a standard surface template. Surfaces are refined using the T2w images. Third, PostFreesurfer converts prior outputs into the HCP-compatible CIFTI data format. In addition, it transforms the volumes to a standard volume template space using ANTs nonlinear registration and the surfaces to a standard surface space via spherical registration. An expert rater, M.B., conducted visual quality control for the anatomical segmentations of each participant and corrected errors in surfaces manually.

### Brain centiles

Individual total volumes of cortical grey matter, whole-brain white matter, and subcortical grey matter together with information on age and sex were entered into the BrainChart tool (https://brainchart.io). The tool uses an out-of-sample maximum likelihood estimator to model site-specific random effects and compute age- and sex-adjusted centiles via the cumulative density function^20^. These centiles reflect each child’s deviation from sex-stratified normative reference models derived from over 120,000 structural brain scans.

### Intellectual ability

All children completed standardized assessments of intellectual ability using the Snijders-Oomen Nonverbal Intelligence Test (SON-R)^24^, yielding standardized IQ scores, and the Verbal Comprehension Scale of the Wechsler Intelligence Scale for Children (WISC)^25^. These tests are well-validated in children with high test-retest reliability (≥0.90) and internal consistency (≥0.96). Children under 6 years of age completed preschool-adapted versions^73^.

### Internalizing and externalizing behaviours

Behavioural problems were assessed using the Strengths and Difficulties Questionnaire (SDQ)^26^. The SDQ is a brief screening questionnaire with good reliability (Cronbach’s alpha = .73). Parents or legal guardians completed the questionnaire answering questions about their child. Internalizing and externalizing scores were computed by summing item responses.

### Socioeconomic status

SES was derived from parental education, occupation, and monthly net income as a multidimensional index score and ranged between 3 and 21^27^.

### Statistical analyses

Statistical analyses were conducted in R version 4.4.1^74^. Separate linear mixed-effects models (LMMs) were fitted for each imaging phenotype, including a random intercept for family to account for the nonindependence of related siblings (Supplementary Table S4). First, estimated marginal means for the CM+ and CM− groups were tested against the age- and sex-normed population mean to assess deviations from typical brain development. Second, group differences were tested by comparing estimated marginal means between CM+ and CM− groups. This complementary within-sample comparison provided a more stringently characterized reference group, given that maltreatment exposure was not systematically assessed in the normative brain-chart datasets. Severity-related variation was examined within the CM+ group only to avoid conflation of exposure presence with graded variation in severity and restricted to levels 2–5 because only one child fell into the lowest category. Additional analyses using LMMs examined group differences in IQ and SDQ scores, as well as associations between brain centiles and IQ and SDQ scores to assess links with functional outcomes. Age and sex were included as covariates in all models to account for residual variation in the sample not fully captured by age- and sex-normed centiles. Multiple comparisons were controlled for using false discovery rate correction^75^ within each predefined family of statistical tests. Standard errors and degrees of freedom for fixed effects were estimated using the Kenward–Roger approximation. Sensitivity analyses additionally tested interactions between maltreatment group and SES to assess moderation by socioeconomic status.

### Reporting summary

Further information on research design is available in the Nature Portfolio Reporting Summary linked to this article.

## Supplementary information

Transparent Peer Review file

Supplementary Information

Description of Supplementary Files

Supplementary Data

Nature Reporting Summary

## Data Availability

The individual-level data underlying this study are not publicly available due to participant privacy and data protection requirements and are available upon reasonable request to the corresponding author. Aggregate numerical source data underlying the main figures are provided as Supplementary Data.

## Code availability

Custom code generated for this project was made openly available at https://github.com/johannesjuliusm/k2h_maltreatment_brain_commsbio2026 and a permanent archive is available on https://zenodo.org/records/22710946^76^. *R*: We made use of the following packages: *broom.mixed* (v0.2.9.7), *charite* (v0.5.0)^77^, *corrplot* (v0.95), *cowplot* (v1.2.0), *dplyr* (v1.2.0), *emmeans* (v2.0.3), *flextable* (v0.9.11), *gghalves* (v0.1.4), *ggplot2* (v4.0.3), *ggpubr* (v0.6.3), *gtsummary* (v2.5.0), *Hmisc* (v5.2-5), *lme4* (v2.0-1), *lmerTest* (v3.2-1), *knitr* (v1.51), *officer* (v0.7.5), *patchwork* (v1.3.2), *performance* (v0.16.0), *psych* (v2.6.5), *purrr* (v1.2.1), *rlang* (v1.2.0), *stringr* (v1.6.0), and *tidyr* (v1.3.2).
