# Supplementary material for: Maltreatment predicts centiles of individual variation from normative brain growth in children
Source: Commun Biol. 2026 Oct 22;9:1465. doi: 10.1038/s42003-026-11143-6 (PMC13736999; doi:10.1038/s42003-026-11143-6)
Supplement: Supplementary file 1 [file 42003_2026_11143_MOESM1_ESM.pdf]

nature portfolio

Peer Review File

Maltreatment Predicts Centiles of Individual Variation from Normative Brain Growth in Children

Corresponding Author: Professor Christine Heim

This file contains all reviewer reports in order by version, followed by all author rebuttals in order by version.

Version 0:

Reviewer comments:

Reviewer #1

(Remarks to the Author)
This study examines whether exposure to childhood maltreatment is associated with deviations from normative brain development by applying the recently developed brain charts to structural magnetic resonance imaging data from children aged 3 to 13 years. The authors report that children with documented maltreatment exposure showed lower age- and sex-adjusted centile scores for cortical grey matter, white matter, and subcortical grey matter volumes, with greater maltreatment severity associated with larger deviations. Lower brain growth centiles were also associated with reduced intellectual ability and increased behavioural difficulties. While the findings are consistent with a substantial body of literature demonstrating associations between early adversity and altered brain development, the primary novelty lies in the application of a normative modelling framework that quantifies individual variation relative to population-based developmental trajectories rather than in the identification of a fundamentally new neurobiological phenomenon.

The study demonstrates that children with maltreatment exposure exhibit lower brain growth centiles than expected based on normative developmental trajectories. This is an important topic with clear relevance for child welfare and public health. However, because the centile scores are derived from volumetric measures that have previously been shown to differ in children exposed to adversity, the principal advance appears to be more methodological than a scientific advance. The centile framework may provide a more standardized and clinically interpretable metric, but it does not substantially alter the current understanding of the relationship between maltreatment and brain development.

The application of normative brain growth centiles is an interesting methodological advance. However, it would be helpful to better understand the added value of this approach relative to conventional volumetric analyses. Specifically, do the centile measures improve prediction of cognitive ability, behavioural outcomes, or clinical functioning compared with just raw brain volumes? Is there evidence that centile scores are more sensitive, clinically interpretable, or better able to identify at-risk individuals than traditional volumetric measures? As a sensitivity analysis, could the authors compare associations obtained using brain growth centiles with those obtained using conventional age- and sex-adjusted volumetric measures? This would aid in determining whether the normative modelling framework provides additional explanatory or predictive value.

Reviewer #2

(Remarks to the Author)
-----1.*What are the major claims of the paper?*-----
This paper derives population centile scores for different brain imaging phenotypes in a sample of children with (CM+) and without maltreatment (CM-). They then compare both groups and their results are indicative of case-control differences between maltreatment exposed and maltreatment-free children. Such differences suggest that, relative to the expected healthy norm, brain tissue of different types in maltreated children appears to be smaller than expected for their age and sex.

Standing question: where do the brains of maltreated children sit relative to the expected healthy norm?

For clarity on my perspective as a reviewer I would like to disclose my name and affiliation: Dr. Sofia Orellana, University of Cambridge, Department of Psychiatry. I would also like to note that I am additionally mentored by Dr. Richard Bethlehem who developed the brain charts on which this paper rests.

My expertise in neuroimaging and childhood maltreatment comes from writing this paper:

Orellana, S. C., Bethlehem, R. A. I., Simpson-Kent, I. L., van Harmelen, A.-L., Vértes, P. E., & Bullmore, E. T. (2024). Childhood maltreatment influences adult brain structure through its effects on immune, metabolic, and psychosocial factors. _Proceedings of the National Academy of Sciences_, _121_(16), e2304704121. https://doi.org/10.1073/pnas.2304704121

--2. A*re these claims novel? Will they interest others in the community and the wider field?* ---

Differences in grey matter structure in maltreated children are widely reported and well stablished in other samples. This paper appears to be the first time that someone compared maltreated children relative to the expected norm for their age and sex by leveraging brain charts.

A population brain growth chart that roughly tells you: where does this individual's value in e.g. grey matter volume sit relative to what is expected on average for their age and sex?. This is analogous to pediatric charts of height growth which can help answer the question: is this child short or tall given what is expected for their age and sex? how much taller/shorter?

Here the question answered is: are there differences relative to the expected norm in different brain tissues when a child has undergone childhood maltreatment?

To my knowledge this is the first time the brain charts framework - with the 100k Bethlehem et al., (2022) sample as a basis of the chart - is applied to childhood maltreatment specifically.

I think this is a good paper that transitions the study of CM away from simple case control and towards comparisons grounded on the expected "developmental status" of an individual.

---3. *If the conclusions are not original, what relevant references establish the prior work?* ---

The conclusions here are novel and the contribution is a valuable one. However, I would like to interrogate the following claim: _"This presents the largest structural neuroimaging study in a sample of children with clinically verified maltreatment exposure to date."_ Given how extensive the literature on MRI-based structural alterations following childhood maltreatment already is (the supplementary table of imaging studies in the Teicher et al. (2016) review alone runs to some 89 pages), I would like to see more clarification on how this is indeed the first time such a study exists. Presumably the novelty rests on a specific combination of features, such as "clinically verified" exposure, sample size, age range, or imaging modality, rather than on being the first study to recruit children for adversity exposure and scan them. Making those qualifying criteria explicit, and ideally benchmarking the sample against a few of the larger existing cohorts, would let readers situate the study's scale within the broader field and would strengthen rather than weaken the contribution. (Something I'd very happy to see!)

I would like to see a little bit more discussed in the same fashion when it comes to the following statement in the discussion:

"Earlier studies observed brain volume differences relating to severe childhood adversity in young adults (Mackes et al., 2020) and adolescents (Busso et al., 2017; Luo et al., 2020; Paquola et al., 2017), with a lack of studies in children."

---4. *Is the work convincing? If not, what further evidence would be required to strengthen the conclusions?* & *Is the statistical analysis appropriate and valid* ?

The work is largely convicing and very competently executed! However the following points should be addressed to strengthen the conclusions:

(A) Any time analyses are reported for the three tissue types in the results, p-values with corrections for multiple comparisons should also be reported and appropriately described in the methods. FDR correction is my choice when dealing with brain analyses as Bonferroni assumes full independence (and brain tissues are not) and is too restrictive. This is necessary in order to assess the robustness of the results. Results should also be discussed accordingly.
(A.1) I think it will be additionally important to discuss robustness to multiple comparisons given correction/moderation for SES. Your results are still likely to stand!

(B) Writing note: throughout the paper the authors refer to centile scores for different brain tissue types as "brain growth centile scores". The scores are obtained *relative* to "brain growth charts" but I believe it is technically incorrect to refer to the centiled tissue scores as "growth scores". No growth is directly being measured here, you are benchmarking against the expected norm and the term "centile" encapsulates that.

(C) If this is not against the journal conventions: Table S1 should be part of the main paper along with a brief text description of the sample characteristics. Please also indicate what test the p-value given on the table is derived from. (Same note on p-values for S2)

(C1) Side note: Thank you for adding Supp Figure 1. This is important and often missing in papers!

(D) For clarity, can you please elaborate a little more in the results how moderation analyses were performed?

(E) You have excellent supplementary information but I don't see this referenced as much in the main text. Please make reference in the main text, when pertinent, to the relevant tables and figures that accompany your main results. For instance when writing about IQ and behaviour you write "There was no evidence for a moderating effect of maltreatment group membership of these associations" and make no reference to where in the supplements this is reported.

(!) An optional suggestion: The data is correctly reported under "Brain growth centiles, intellectual ability, and behavioural problems". The overall flow of the paper would be improved by adding a matrix of correlation values between centile scores and IQ/externalising symptoms as a panel figure, along with a couple of scatterplots for the most significant association. You can heat-map color the matrix entries by degree of correlation and also visually indicate the significance of the association, and discuss the possible meaning of the strongest associations vs those that are weakest.

---5. *(Subjective) Do you think the paper will influence thinking in the field?* ----
I think this was a necessary first step towards moving CM and brain structure studies into a methodological framework with real neurodevelopmental benchmarks. Its potential lies in nudging the field towards a more novel set of methodological approaches.

---6. *Are there any further questions or concerns about the paper you want to raise?* ----

(A) For readability I would like more detail about the methods employed described within the results section. For instance:

"There were significant differences between CM+ and CM- groups in growth centiles of all three tissue types (Fig. 1): total grey matter volume, $t(259) = 3.35$, Cohen’s $d = 0.4$ (small effect), $p < 0.001$; total white matter volume, $t(249) = 4.38$, Cohen’s $d = 0.52$ (medium effect), $p < 0.001$; total subcortical grey matter volume, $t(232) = 2.54$, Cohen’s $d = 0.31$ (small effect), $p = 0.012$)."

I understand you report t-tests within the methods but it is worth specifying in the results text, at least once, when t-tests are employed and when linear regression is employed.

(B) In figure 1 there is the line: "Colour gradient from yellow to dark red represents maltreatment severity." but there is no colour gradient.

(C) I appreciate the addition of the following description of how CM scores were derived: _"Maltreatment severity scores were computed as the sum of all severity ratings of each reported incident of maltreatment according to the Maltreatment Classification System and thus ranged from 0 to 5."_ Could you please rephrase this sentence for readability? As currently worded, "the sum of all severity ratings of each reported incident" implies that a separate score is assigned per maltreatment type (each potentially ranging from 0 to 5) and that these subscores are then summed. From what I understand, this is not the procedure you followed. Clarifying exactly what is being summed, and over what, would resolve the ambiguity

(D) In the beginning of the discussion you write "indicating greatest neurodevelopmental aberrations" - I don't think you can or should describe these as aberrations. (1) Variation in centile values towards the lower end are observed in both samples and (2) the term aberration indicates some sort of malformation - which is not something that these centile measures can be interpreted as (given this data).

--7. *Could another researcher reproduce the work given the level of detail provided*? ---

Overall there is a very good level of detail in this paper except where indicated above. If the additional detail requested is provided this should be replicable.

Version 1:

Reviewer comments:

Reviewer #1

(Remarks to the Author)
I thank the authors for their thoughtful and thorough responses to my comments. The manuscript has been substantially revised, and I particularly appreciate the additional analyses comparing normative centile measures with conventional volumetric measures. These analyses are helpful and provide important context for understanding the contribution of the normative modelling approach.

I have only a few remaining comments, which primarily concern interpretation and framing rather than the need for substantial additional analyses.

My primary comment in the original review concerned the added value of normative centiles relative to conventional volumetric analyses. I appreciate that the authors directly addressed this question with additional sensitivity analyses. Importantly, however, these analyses suggest that the incremental predictive/explanatory value of centiles is relatively modest. For example, discrimination of maltreatment status was weak for both approaches (AUCs approximately 0.57–0.65), and centiles did not improve prediction over raw volumes. Similarly, associations with cognitive and behavioural measures were generally comparable across the two approaches, although there were some instances in which centiles appeared somewhat more sensitive.
I don't view this as a weakness that requires further analysis. Rather, I think it provides an opportunity to more clearly define the contribution of the study. The normative framework provides an intuitive, population-referenced way of expressing individual variation, but the present findings do not demonstrate a substantial improvement in explanatory or predictive performance over conventional volumetric approaches. I would encourage the authors to acknowledge this more explicitly in the manuscript and frame the contribution accordingly.

Relatedly, I would suggest somewhat tempering statements regarding the clinical implications of the centile measures. I agree that expressing brain volumes relative to age- and sex-normative distributions is intuitive and potentially clinically interpretable. However, the present study does not establish clinical utility or demonstrate that these measures can reliably identify individual children at risk. Indeed, predictive discrimination was relatively modest.

I therefore suggest distinguishing more clearly between interpretability relative to a normative population and demonstrated clinical utility. The former is a strength of this approach; the latter will require further prospective validation.

I also encourage the authors to carefully review references to altered or deviant "developmental trajectories." The normative centiles are derived from age-related developmental reference curves, but the present study itself is cross-sectional. Thus, the data demonstrate that children occupy different positions relative to age- and sex-normative distributions; they do not directly demonstrate altered within-child trajectories of brain development. The authors appropriately acknowledge the cross-sectional design as a limitation, and I think ensuring that this distinction is maintained consistently throughout the manuscript would strengthen the interpretation.

Finally, the statement that "severity of maltreatment was associated with lower brain centiles" appears somewhat stronger than the corrected analyses support. Following FDR correction, the continuous severity associations with cGM, WM, and sGM centiles were not statistically significant (p_{FDR} = .066, .066, and .172, respectively), although children with higher severity ratings showed lower centiles relative to the population norm. I suggest revising the abstract and related statements so that they accurately reflect this more nuanced result.

Reviewer #2

(Remarks to the Author)
The authors have done an excellent job addressing my comments. I recommend accepting this manuscript. I only have a couple of observations to improve the manuscript should the authors be happy to add them. I don't expect any responses back.

1. The response to my comments on the last paragraph of page 9 are a strong argumentation for the novelty of the dataset. It is worth folding this into the introduction or discussion to highlight that.

2.This is just a minor aesthetic comment: experiment with increasing the font size within the matrix entries for figure 3.

Congratulations!

Open Access This Peer Review File is licensed under a Creative Commons Attribution 4.0 International License, which permits use, sharing, adaptation, distribution and reproduction in any medium or format, as long as you give appropriate credit to the original author(s) and the source, provide a link to the Creative Commons license, and indicate if changes were made.
In cases where reviewers are anonymous, credit should be given to 'Anonymous Referee' and the source.
The images or other third party material in this Peer Review File are included in the article's Creative Commons license, unless indicated otherwise in a credit line to the material. If material is not included in the article's Creative Commons license and your intended use is not permitted by statutory regulation or exceeds the permitted use, you will need to obtain permission directly from the copyright holder.
To view a copy of this license, visit https://creativecommons.org/licenses/by/4.0/

Maltreatment Predicts Centiles of Individual Variation from Normative Brain Growth in Children
COMMSBIO-26-4414-T
Mohn et al.
Revised Manuscript

July 9, 2026

POINT-BY-POINT RESPONSE TO REVIEWERS

We sincerely thank the editors and reviewers for appreciating the scientific significance of our study and for showing enthusiasm for our results. We greatly appreciate the time and expertise invested in reviewing our work and are grateful for the opportunity to submit a substantially revised version of the manuscript.

We found the reviewers' comments to be extremely helpful and have addressed all comments and revised the manuscript accordingly. We believe that these revisions have significantly strengthened both the clarity and the scientific contribution of the manuscript. Below, we provide detailed point-by-point responses to each item.

Responses to Reviewer 1 (R1)

We sincerely thank R1 for the thoughtful evaluation of the theoretical implications our work and for discussing the potential value of applying normative brain growth charts to the study of the effects of maltreatment exposure on brain development in children. We appreciate the constructive suggestions, which have helped us to improve the framework and critical discussion of our methods and results in the revised manuscript.

1. **Comment #1: The centile framework may provide a more standardized and clinically interpretable metric, but it does not substantially alter the current understanding of the relationship between maltreatment and brain development. The application of normative brain growth centiles is an interesting methodological advance. However, it would be helpful to better understand the added value of this approach relative to conventional volumetric analyses.**

Specifically, do the centile measures improve prediction of cognitive ability, behavioural outcomes, or clinical functioning compared with just raw brain volumes? Is there evidence that centile scores are more sensitive, clinically interpretable, or better able to identify at-risk individuals than traditional volumetric measures? As a sensitivity analysis, could the authors compare associations obtained using brain growth centiles with those obtained using conventional age- and sex-adjusted volumetric measures? This would aid in determining whether the normative modelling framework provides additional explanatory or predictive value.

We thank the reviewer for his thoughtful assessment of the value of our study methods and the additional insightful comments and suggestions they provided.

a) "**It does not substantially alter the current understanding of the relationship between maltreatment and brain development".** It is correct that the primary contribution of our study is the application of normative modelling on a known relationship. Hence, as stated by R1, in principle, our results do not alter our current understanding of the association between maltreatment exposure and brain development. Instead, our results *confirm* an impact of maltreatment on brain development using an improved methodology (as also stated by Reviewer 2, see below):
b) **"It would be helpful to better understand the added value of this approach relative to conventional volumetric analyses".** Unlike conventional volumetric measures, centiles place an individual's brain phenotype in the context of a large normative reference population while accounting for age and sex (Bethlehem et al., 2022). Analogous to paediatric growth charts for height and weight, they allow clinicians and researchers to determine whether a child's brain volume falls within the expected range for their developmental stage or deviates substantially from it, providing an intuitive and standardized measure of neurodevelopmental status. For example, in the present study, children with maltreatment exposure ranked, on average, around the 40th percentile, corresponding to a 10-percentile downward shift relative to the population. In the revised manuscript, we now included these comparisons against the population mean in the Results and have also added them to a revised version of Figure 1 (also see below, Response to R2, Comment 5B).

Expressing results in this way provides an immediately interpretable measure of neurodevelopmental status. Furthermore, recent work in which our group was involved demonstrates the utility of growth charts for identifying individual patients (Zughayyar et al., 2025), providing evidence that centile scores are more sensitive and supporting their potential clinical applicability.

c) **Specifically, do the centile measures improve prediction of cognitive ability, behavioural outcomes, or clinical functioning compared with just raw brain volumes? Is there evidence that centile scores are more sensitive, clinically interpretable, or better able to identify at-risk individuals than traditional volumetric measures? As a sensitivity analysis, could the authors compare associations obtained using brain growth centiles with those obtained using conventional age- and sex-adjusted volumetric measures?** We appreciate this excellent suggestion of R1. In the manuscript we show that centile scores are associated with various outcomes. Here now we provide the requested sensitivity analysis, comparing the predictive value of raw volumetric measures versus centile scores.

First, we explored group differences between CM+ and CM– groups in raw brain volumes, adjusting for age and sex (Figure A). There were significant differences between groups in cortical grey matter volumes (β = -2.13, 95 % CI [-3.29, -0.96], $p < 0.001$) and white matter volumes (β = -2.50, 95 % CI [-3.67, -1.32], $p < 0.001$), but not in subcortical grey matter volumes (β = -0.05, 95 % CI [-0.26, 0.15], p = 0.609). Because a small number of individuals with relatively large subcortical volumes exerted a disproportionate influence on the group means, group differences only became apparent when expressing subcortical volumes as normative centiles (compare Figure A to Figure 1, below in response to R2).

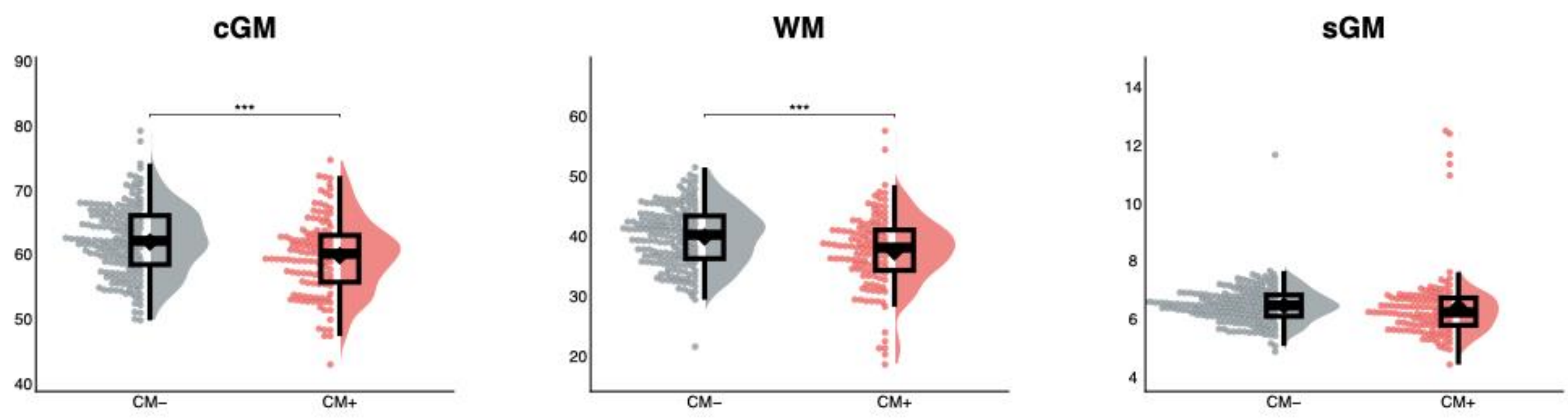

Figure A. Maltreatment-related differences in raw brain volumes. cGM, cortical grey matter, WM, white matter; sGM, subcortical grey matter. Asterisks denote statistical significance: p < 0.05 *, p < 0.01 **, p < 0.001 ***. Only significant differences are shown. Note that y axes are scaled in units of 10,000 mm^3 (10 ml).

Next, to test the predictive ability of raw volumes and brain centiles, we conducted exploratory binary logistic regressions predicting maltreatment group status (CM+ vs. CM–) from brain measures. The models indicated that both brain volumes and centiles were significant predictors of group membership, however their discriminative ability was weak, with area under the receiver operating characteristic curves (AUC) ranging from 0.57 to 0.65 (see Figure B). Centiles did not improve the prediction over raw volumes. The full model parameters are listed below.

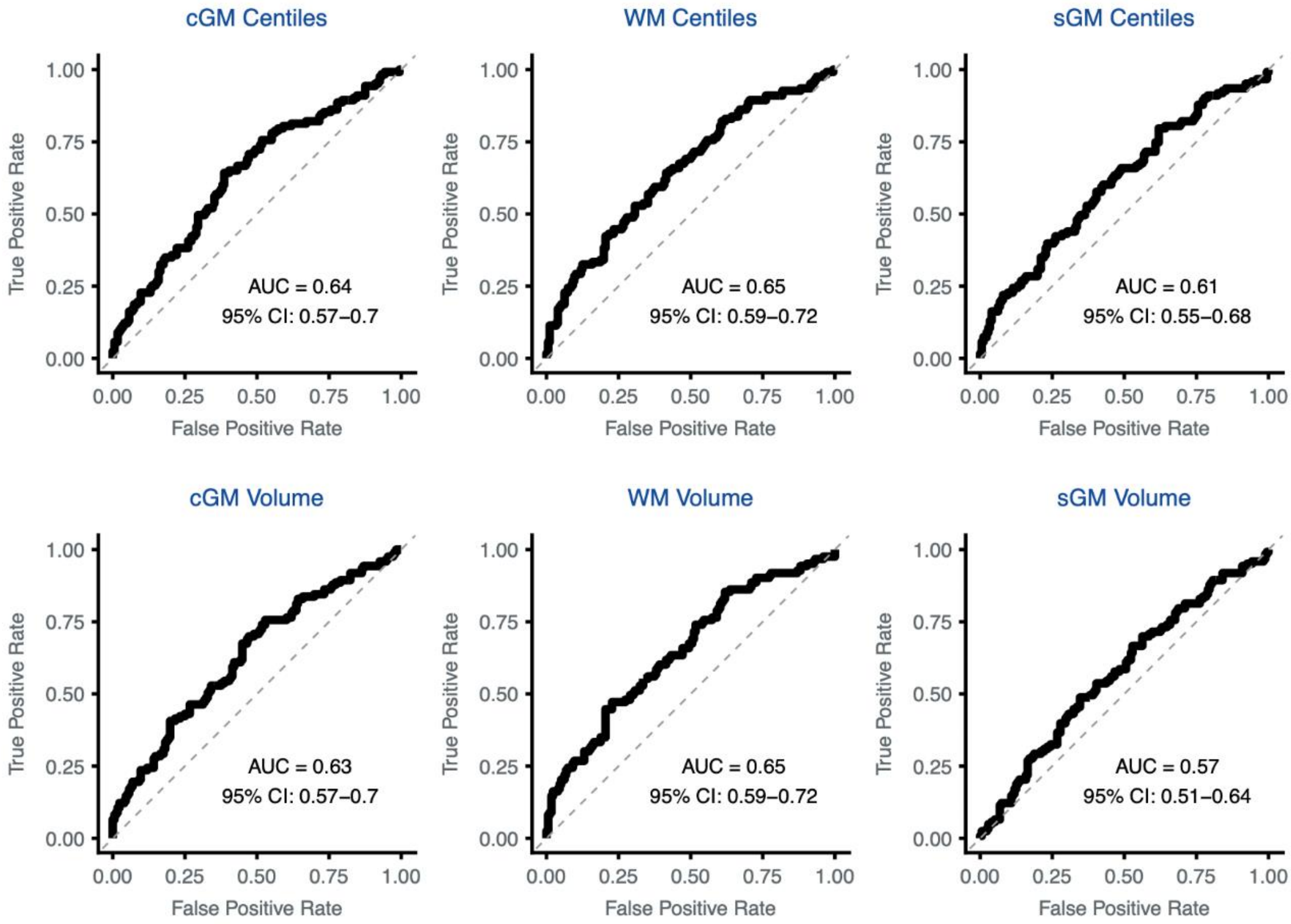

Figure B. Receiver operating characteristic curves for predictions of maltreatment group status from brain measures. Top row: Discriminative ability of centile scores for the prediction of binary maltreatment group status. Bottom row: Discriminative ability of raw brain volumes for the same prediction.

Full model parameters of binary logistic regressions predicting maltreatment group status from brain measures, controlling for residual effects of age and sex:

cGM centiles: β = -1.46, SE = 0.42, z = -3.48, p = 0.001; OR = 0.23, 95% CI [0.10, 0.52]; AUC = 0.64, 95% CI [0.57, 0.70]; WM centiles: β = -1.97, SE = 0.49, z = -4.05,

$p < 0.001$; OR = 0.14, 95% CI [0.05, 0.36]; AUC = 0.65, 95% CI [0.59, 0.72]; sGM centiles: β = -1.36, SE = 0.52, z = -2.63, p = 0.009; OR = 0.26, 95% CI [0.09, 0.70]; AUC = 0.61, 95% CI [0.55, 0.68].

cGM volumes: β = -0.09, SE = 0.02, z = -3.47, p = 0.001; OR = 0.92, 95% CI [0.87, 0.96]; AUC = 0.63, 95% CI [0.57, 0.70]; WM volumes: β = -0.10, SE = 0.03, z = -3.87, $p < 0.001$; OR = 0.90, 95% CI [0.86, 0.95]; AUC = 0.65, 95% CI [0.59, 0.72]; sGM volumes: β = -0.07, SE = 0.14, z = -0.51, p = 0.608; OR = 0.93, 95% CI [0.69, 1.21]; AUC = 0.57, 95% CI [0.51, 0.64].

Finally, we repeated analyses testing associations of brain measures with intelligence (IQ scores) and with affective symptoms (SDQ scores). For comparability of coefficients, we standardized both raw brain volumes and centile scores so that means were zero and coefficients express the change in the response variable for every 1 SD change in the brain variable.

Associations of brain centiles with non-verbal IQ scores (SON-R).

cGM centiles: β = 5.84, SE = 0.97, $p < 0.001$; adj. $R^2 = 0.11$;

cGM volumes: β = 6.29, SE = 1.01, $p < 0.001$; adj. $R^2 = 0.10$.

WM centiles: β = 4.17, SE = 1.00, $p < 0.001$; adj. $R^2 = 0.05$;

WM volumes: β = 3.67, SE = 1.10, $p < 0.001$; adj. $R^2 = 0.03$.

sGM centiles: β = 3.83, SE = 0.99, $p < 0.001$; adj. $R^2 = 0.05$;

sGM volumes: β = 3.30, SE = 1.01, p = 0.002; adj. $R^2 = 0.03$.

Associations of brain centiles with verbal IQ scores (WISC-Verbal).

cGM centiles: β = 3.72, SE = 1.01, $p < 0.001$; adj. $R^2 = 0.03$;

cGM volumes: β = 3.89, SE = 1.20, p = 0.001; adj. $R^2 = 0.03$.

WM centiles: β = 3.03, SE = 1.01, p = 0.005; adj. $R^2 = 0.02$;

WM volumes: β = 2.01, SE = 1.18, p = 0.089; adj. $R^2 = 0.002$.

sGM centiles: β = 3.26, SE = 1.05, p = 0.002; adj. $R^2 = 0.02$;

sGM volumes: β = 2.37, SE = 1.16, p = 0.042; adj. $R^2 = 0.006$.

Associations of brain centiles with internalizing (SDQ).

cGM centiles: β = -0.59, SE = 0.20, p = 0.004; adj. $R^2 = 0.03$;

cGM volumes: β = -0.61, SE = 0.23, p = 0.009; adj. R^2 = 0.03.

WM centiles: β = -0.61, SE = 0.21, p = 0.004; adj. R^2 = 0.03;

WM volumes: β = -0.60, SE = 0.24, p = 0.011; adj. R^2 = 0.02.

sGM centiles: β = -0.43, SE = 0.21, p = 0.041; adj. R^2 = 0.02;

sGM volumes: β = -0.34, SE = 0.25, p = 0.172; adj. R^2 = 0.007.

Associations of brain centiles with externalizing (SDQ).

cGM centiles: β = -0.95, SE = 0.24, p < 0.001; adj. R^2 = 0.04;

cGM volumes: β = -1.18, SE = 0.27, p < 0.001; adj. R^2 = 0.05.

WM centiles: β = -0.72, SE = 0.26, p = 0.005; adj. R^2 = 0.02;

WM volumes: β = -0.75, SE = 0.29, p = 0.009; adj. R^2 = 0.01.

sGM centiles: β = -0.67, SE = 0.25, p = 0.009; adj. R^2 = 0.01;

sGM volumes: β = -0.28, SE = 0.30, p = 0.35; adj. R^2 = 0.00.

In response to R1, we have further refocused the Introduction by condensing background material on neuroplasticity and the association between childhood maltreatment and psychopathology. This has allowed us to expand the discussion of normative brain centiles, including their rationale, interpretability, and potential advantages over conventional volumetric measures, thereby making the central contribution of the study clearer from the outset (Introduction, tc-p. 3–4):

"Recently, brain growth charts, derived from over120,000 brain scans, have become available and provide a novel quantitative framework for situating individual brain volumes relative to age- and sex-matched norms (Bethlehem et al., 2022). Analogous to paediatric growth charts for height and weight, they allow researchers and clinicians to determine whether a child's brain volume falls within the expected range for their developmental stage or deviates substantially from it, providing an intuitive and standardized measure of neurodevelopmental status (Zughayyar et al., 2025). Brain centiles demonstrate longitudinal stability within subjects and robustness to variations in data acquisition across studies (Bethlehem et al., 2022). As a result, centile scores provide a standardized and directly clinically interpretable metric of individual variation in brain structure. Importantly, brain charts extend cross-sectional case-control analyses by placing group differences within a shared developmental reference that offers insight into the magnitude, direction, and heterogeneity of deviations from typical development (Marquand et al., 2016)."

We also added a discussion of the conceptual advance of our study to the Discussion (tc-p. 7–8):

"The present findings extend a growing body of evidence linking childhood maltreatment to altered brain development (Malave et al., 2022; Nelson et al., 2025; Vaidya et al., 2024) by observing widespread deviations from normative developmental trajectories rather than isolated differences in individual brain regions or tissue types. On average, children with confirmed maltreatment ranked lower across cortical grey matter, subcortical grey matter, cortical surface area, and white matter, indicating broadly reduced brain development. These findings are consistent with previous large-scale studies reporting smaller global and regional brain volumes following childhood maltreatment (Busso et al., 2017; Holz et al., 2023; Joseph et al., 2023; Mackes et al., 2020; Wang et al., 2026). We extend this literature by quantifying deviations relative to normative brain growth charts. Framing brain development in terms of centiles provides an intuitive measure of individual neurodevelopmental variation that facilitates comparisons across tissue types, developmental stages, phenotypes, and studies. Beyond characterizing average group differences, normative brain charts identify children with the most pronounced neurodevelopmental deviations relative to population expectations, providing a framework for investigating why some children show marked neurodevelopmental impacts following maltreatment whereas others remain comparatively resilient."

If requested by R1, we are happy to include these analyses in the Supplement.

Responses to Reviewer 2 (R2)

We thank R2 for her supportive and positive evaluation of our study. We appreciate the many thoughtful comments that have been a major help for us to further improve the manuscript. We further note that many aspects raised by R1 on the utility and advantage of using centile-based normative brain charts are stated in the comments of R2. We are grateful that R2 clearly stated the advantage of using brain charts. In the following, we respond to each comment of R2.

1. **Comment #1: Differences in grey matter structure in maltreated children are widely reported and well stablished in other samples. This paper appears to be the first time that someone compared maltreated children relative to the expected norm for their age and sex by leveraging brain charts. A population**

brain growth chart that roughly tells you: where does this individual's value in e.g., grey matter volume sit relative to what is expected on average for their age and sex? This is analogous to paediatric charts of height growth which can help answer the question: is this child short or tall given what is expected for their age and sex? how much taller/shorter?
Here the question answered is: are there differences relative to the expected norm in different brain tissues when a child has undergone childhood maltreatment? To my knowledge this is the first time the brain charts framework—with the 100k Bethlehem et al., (2022) sample as a basis of the chart—is applied to childhood maltreatment specifically. I think this is a good paper that transitions the study of CM away from simple case control and towards comparisons grounded on the expected "developmental status" of an individual.

We sincerely thank R2 for these very encouraging comments and for emphasizing the novelty of applying the normative brain charts framework to the study of childhood maltreatment. We are delighted that R2 considers our approach a meaningful and important advance that improves interpretation of individual neurodevelopment relative to normative developmental trajectories rather than relying solely on traditional case-control comparisons. We fully share R2's view that this framework has the potential to provide a developmentally informed perspective on the neurobiological consequences of childhood maltreatment. To further emphasize this contribution, we have revised the Introduction and Discussion (see above, Response to R1) to articulate the conceptual advantages of normative modelling and its potential applications more clearly in future research.

2. **Comment #2: The conclusions here are novel and the contribution is a valuable one. However, I would like to interrogate the following claim: *"This presents the largest structural neuroimaging study in a sample of children with clinically verified maltreatment exposure to date."* Given how extensive the literature on MRI-based structural alterations following childhood maltreatment already is (the supplementary table of imaging studies in the (Teicher et al., 2016) review alone runs to some 89 pages), I would like to see more clarification on how this is indeed the first time such a study exists. Presumably the novelty rests on a specific combination of features, such as "clinically verified" exposure, sample**

size, age range, or imaging modality, rather than on being the first study to recruit children for adversity exposure and scan them. Making those qualifying criteria explicit, and ideally benchmarking the sample against a few of the larger existing cohorts, would let readers situate the study's scale within the broader field and would strengthen rather than weaken the contribution. (Something I'd be very happy to see!).

I would like to see a little bit more discussed in the same fashion when it comes to the following statement in the discussion: "Earlier studies observed brain volume differences relating to severe childhood adversity in young adults (Mackes et al., 2020) and adolescents (Busso et al., 2017; Luo et al., 2020; Paquola et al., 2017), with a lack of studies in children."

We sincerely thank R2 for highlighting this point. We agree that our original wording was too superlative and the explicit features that make our study unique were not stated or analysed. We understand that R1 would like to see such an analysis (see above).

We changed the strength statement of our study in the Limitations section accordingly (Discussion, tc-p. 10):

"This presents the largest structural neuroimaging study in a sample of children with clinically verified maltreatment exposure to date. While we used well-validated assessment tools and state-of-the-art processing for developmental MRI data with extensive manual quality control, this study is not without limitations."

Furthermore, there is a multitude of studies on neurostructural changes in children and adults as a function of maltreatment exposure (Teicher et al., 2016). These studies mostly focus on regional brain volume changes, such as hippocampus, and they have mostly been conducted in adults. There are far less studies assessing global brain volumes, and many of these have been conducted in adults with maltreatment exposure assessed retrospectively by self-report or questionnaire. One small study of our group (Joseph et al., 2023) studied the impact of maltreatment severity on global brain volume in 49 children, but this study did not include a control group. Very recently, Wang et al., (2026) applied normative modelling in 3,711 participants from the ENIGMA Consortium, including ~350 children and adolescents under 18 years. This study retrospectively assessed maltreatment through self-report on the Childhood Trauma Questionnaire (Bernstein et al., 2003) and found no effects of

maltreatment in this age group. We provide a comparably sized study of children aged 3–13 years that were recruited from child welfare and protection services and maltreatment was assessed by trained clinicians using the comprehensive and well-validated Maltreatment Classification Interview (Cicchetti et al., 2003). Moreover, we imaged children using a child-adapted MRI protocol and state-of-the-art neuroimaging processing optimized for children and youth which we obtained from the authors of the ABCD study. Our study thus presents the largest sample of children with clinically assessed and validated maltreatment exposure and high-quality neuroimaging data to date.

3. **Comment #3 (A): Any time analyses are reported for the three tissue types in the results, p-values with corrections for multiple comparisons should also be reported and appropriately described in the methods. FDR correction is my choice when dealing with brain analyses as Bonferroni assumes full independence (and brain tissues are not) and is too restrictive. This is necessary in order to assess the robustness of the results. Results should be discussed accordingly.**
   It will be additionally important to discuss robustness to multiple comparisons given correction/moderation for SES.

We sincerely thank R2 for raising this crucial point and we acknowledge that multiple models were estimated across tissue types, exposures, and outcomes. Our initial rationale was to examine a set of *a priori* questions across related neuroanatomical measures and outcomes. Accordingly, we focused on consistency of effect direction and patterns across tissue types rather than on isolated *p*-values and, in the first version of the manuscript, therefore did not apply formal multiple-testing corrections. However, after further consideration, we now fully agree with R2 that correction for multiple comparisons is crucial.

We now include FDR-corrected *p*-values alongside the raw *p*-values for each family of tests that were run in the Results. We hope that this will allow researchers to judge the robustness of our results. The main conclusions of the paper were not affected by this change, but analyses of maltreatment severity were less conclusive.

4. **Comment #3 (B): Writing note: throughout the paper the authors refer to centile scores for different brain tissue types as “brain growth centile scores”. The scores are obtained *relative* to “brain growth charts” but I believe it is technically**

incorrect to refer to the centiled tissue scores as "growth scores". No growth is directly being measured here, you are benchmarking against the expected norm and the term "centile" encapsulates that.

We truly thank R2 for noting this important point and have accordingly changed the terminology throughout the manuscript. We agree that the term "centile" sufficiently encapsulates the benchmarking of a measure against a population norm and indicates whether a child is above, below or at typical growth.

5. **Comment #3 (C): If this is not against the journal conventions: Table S1 should be part of the main paper along with a brief text description of the sample characteristics. Please also indicate what test the p-value given on the table is derived from. (Same note on p-values for S2). Side note: Thank you for adding Supplementary Figure 1. This is important and often missing in papers!**

We thank R2 for this helpful suggestion and for the positive comment regarding Supplementary Figure 1. We also very much appreciate that R2 states that the distribution of socioeconomic status (SES) is rarely reported in papers on maltreatment effects, which is particularly problematic given the convolution of these variables. To fully meet R2's suggestion, we moved Tables S1 and S2 to the main text. In addition, we have clarified in the table legends which statistical tests were used to derive the reported *p*-values.

6. **Comment #3 (D): For clarity, can you please elaborate a little more in the results how moderation analyses were performed?**

We thank R2 for this suggestion. We fully agree that the original Results section did not sufficiently describe the moderation analyses. We have now added a brief description of the statistical models at the beginning of this section, specifying that moderation was tested using linear mixed-effects models including an interaction term between maltreatment group and socioeconomic status (adjusting for age, sex and a random intercept for family clustering). This additional information provides readers with the necessary context while avoiding repetition of the detailed statistical procedures described in the Methods (Results, tc-p. 6–7):

"We tested whether SES moderated the associations between maltreatment exposure and brain centiles by including an interaction term of maltreatment group status and SES in the main linear mixed-effects models while controlling for age, sex and family clustering."

7. **Comment #3 (E): You have excellent supplementary information, but I don't see this referenced as much in the main text. Please refer in the main text, when pertinent, to the relevant tables and figures that accompany your main results. For instance, when writing about IQ and behaviour you write "There was no evidence for a moderating effect of maltreatment group membership of these associations" and make no reference to where in the supplements this is reported.**
   An optional suggestion: The data is correctly reported under "Brain growth centiles, intellectual ability, and behavioural problems". The overall flow of the paper would be improved by adding a matrix of correlation values between centile scores and IQ/externalizing symptoms as a panel figure, along with a couple of scatterplots for the most significant association. You can heat-map colour the matrix entries by degree of correlation and also visually indicate the significance of the association and discuss the possible meaning of the strongest associations vs those that are weakest.

We thank R2 for stating that we have excellent supplementary information. We further thank R2 for the helpful suggestions in referencing the information. We have now added explicit references throughout the main text to the relevant Supplementary Tables and Figures where appropriate, including for the analyses relating to intellectual ability and behavioural outcomes.

We also appreciate the suggestion to include a correlation matrix. We agree that this would be a useful visualization of the results and have added a new panel figure showing the correlation matrix (Figure 3 in the manuscript).

8. **Comment #4: I think this study was a necessary first step towards moving CM and brain structure studies into a methodological framework with real neurodevelopmental benchmarks. Its potential lies in nudging the field towards a more novel set of methodological approaches.**

We thank R2 for these thoughtful and encouraging remarks and for explicitly stating that this study is a "necessary step". This is also important in light of R1's concerns. We are pleased that R2 recognizes the potential of this work to advance the field by embracing a normative neurodevelopmental framework for studying the effects of childhood maltreatment.

9. **Comment #5 (A): For readability I would like more detail about the methods employed described within the results section. For instance: "There were significant differences between CM+ and CM- groups in growth centiles of all three tissue types (Fig. 1): total grey matter volume, t(259) = 3.35, Cohen's d = 0.4 (small effect), p < 0.001; total white matter volume, t(249) = 4.38, Cohen's d = 0.52 (medium effect), p < 0.001; total subcortical grey matter volume, t(232) = 2.54, Cohen's d = 0.31 (small effect), p = 0.012)." I understand you report t-tests within the Methods, but it is worth specifying in the Results text, at least once, when t-tests are employed and when linear regression is employed.**

We thank R2 for this helpful suggestion. We agree that the original manuscript could have been clearer in the description of analyses employed. We have therefore substantially revised the statistical analysis section in the Methods to provide a comprehensive description of all analyses performed. More importantly, we have carefully re-considered our analytic strategy and updated our two-step approach (group comparisons in the full sample with sensitivity analyses in a reduced sample of non-related individuals) to linear mixed-effects modelling with a random intercept for family structure. This approach allows us to reduce the number of models run and uses the full sample information while controlling for the non-independence of data from children of the same family (Methods, tc-p. 14):

"Statistical analyses were conducted in R version 4.4.1 (R Core Team, 2024). Separate linear mixed-effects models (LMMs) were fitted for each imaging phenotype, including a random intercept for family to account for the nonindependence of related siblings (SI Appendix, Table S1). First, estimated marginal means for the CM+ and CM– groups were tested against the age- and sex-normed population mean to assess deviations from typical brain development. Second, group differences were tested by comparing estimated marginal means. Severity-related variation was examined within the CM+ group only to avoid conflation of exposure presence with graded variation in severity and restricted to levels 2–5 because only one child fell into the lowest category. Additional analyses using

LLMs examined group differences in IQ and SDQ scores, as well as associations between brain centiles and IQ and SDQ scores to assess links with functional outcomes. Age and sex were included as covariates in all models to account for residual variation in the sample not fully captured by age- and sex-normed centiles. Multiple comparisons were controlled for using false discovery rate correction (Benjamini & Hochberg, 1995) within each predefined family of statistical tests. Inference for fixed effects were estimated using the Kenward–Roger approximation for degrees of freedom. Sensitivity analyses additionally tested interactions between maltreatment group and income-to-needs to assess moderation by socioeconomic status."

10. Comment #5 (B): In Figure 1 there is the line: "Colour gradient from yellow to dark red represents maltreatment severity.", but there is no colour gradient.

We thank the reviewer for pointing out this oversight. The figure legend inadvertently contained wording from an earlier version of the figure. We sincerely apologize for this oversight. We have corrected the legend to accurately describe the final figure. Furthermore, we have updated Figure 1 to include both the group differences between the children with maltreatment exposure and un-exposed children, as well as comparisons of each group with the population mean (50th percentile).

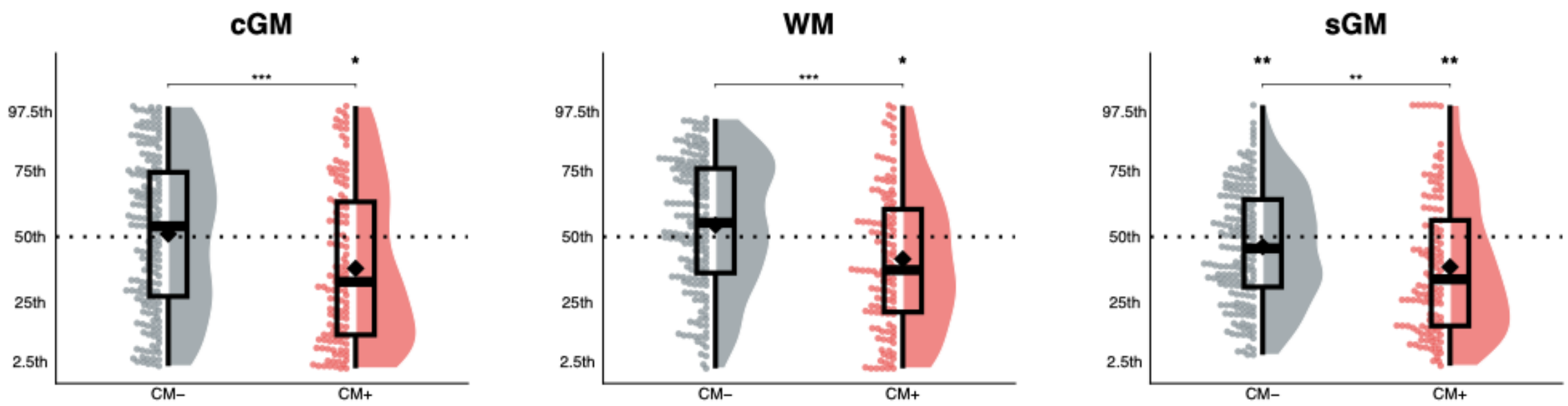

Figure 1. Maltreatment-related differences in global brain centiles. Estimated marginal mean contrasts from linear mixed-effects models adjusted for age, sex and family clustering. cGM, cortical grey matter; WM, total white matter; sGM, subcortical grey matter. Box plot elements: The center line represents the median; box limits denote the upper and lower quartiles (Q3 and Q1), spanning the interquartile range (IQR); whiskers extend to the smallest and largest values within 1.5 × IQR beyond the quartiles. Diamonds denote group means. P values were adjusted for multiple comparisons using the false discovery rate. Asterisks denote statistical significance: $p < 0.05$ *, $p < 0.01$ **, $p < 0.001$ ***. Asterisks above group distributions indicate differences from the population mean; asterisks with brackets compare groups.

11. Comment #5 (C): I appreciate the addition of the following description of how CM scores were derived: *"Maltreatment severity scores were computed as the sum*

of all severity ratings of each reported incident of maltreatment according to the Maltreatment Classification System and thus ranged from 0 to 5."

Could you please rephrase this sentence for readability? As currently worded, "the sum of all severity ratings of each reported incident" implies that a separate score is assigned per maltreatment type (each potentially ranging from 0 to 5) and that these sub-scores are then summed. From what I understand, this is not the procedure you followed. Clarifying exactly what is being summed, and over what, would resolve the ambiguity.

We thank the reviewer for identifying this editing oversight. Indeed, as correctly described in the manuscript, each maltreatment type experienced by a child was rated in severity from mild (1) to life-threatening (5) (and zero representing "none" for children without maltreatment). However, we have corrected the description to clarify that the maltreatment severity score corresponds to the maximum severity rating across all recorded incidents of maltreatment, not its sum. For example, if a child had experienced physical abuse rated severe (4) and emotional neglect rated mild (1), then the total maximum severity was 4. This is the value that was used in analyses of maltreatment severity. We believe the revised description of the maltreatment variables in Methods removes any ambiguity (Methods, tc-p. 12–13):

"Each incident was assigned to one of the five maltreatment subtypes and rated for severity from mild (1) to life-threatening (5). Following prior work (Barnett et al., 1993), exposure was defined as severity ≥ 2, except for physical and sexual abuse, for which any verified occurrence was sufficient. Children with at least one exposure were classified as "verified childhood maltreatment" (CM+). Maltreatment severity was calculated as the maximum severity rating across all five subscales."

12. **Comment #5 (D): In the beginning of the Discussion, you write "indicating greatest neurodevelopmental aberrations" – I don't think you can or should describe these as aberrations. (1) Variation in centile values towards the lower end are observed in both samples and (2) the term aberration indicates some sort of malformation—which is not something that these centile measures can be interpreted as (given this data).**

We sincerely thank R2 for this thoughtful comment and agree that the term "aberrations" is too strong and may imply pathological alterations that cannot be inferred from our data. We have, therefore, replaced this wording with more neutral language, referring instead to greater deviations from normative brain centiles or greater deviations from normative neurodevelopmental trajectories throughout the manuscript, which more accurately reflects the interpretation supported by our findings.

13. **Comment #6: Overall there is a very good level of detail in this paper except where indicated above. If the additional detail requested is provided this work should be replicable.**

We thank the reviewer for this encouraging comment. With the helpful comments made above, we have further improved the methodological descriptions throughout the manuscript in response to the reviewer's helpful suggestions and would like to point out once more that we included a Code Availability statement in the original manuscript indicating that the analysis code will be made publicly available upon publication to facilitate reproducibility.

We would like to once again express our sincere gratitude to the Editor and both reviewers for their thoughtful, constructive, and encouraging feedback. Their comments have been invaluable in improving the quality and clarity of our manuscript. We believe that all concerns have been thoroughly addressed, and the revised manuscript reflects these improvements.

References

Barnett, D., Manly, J. T., & Cicchetti, D. (1993). Defining child maltreatment: The interface between policy and research. In D. Cicchetti & S. L. Toth (Eds), *Child Abuse, Child Development and Social Policy* (Vol. 8, pp. 7–73). Ablex Publishing.

Benjamini, Y., & Hochberg, Y. (1995). Controlling the False Discovery Rate: A Practical and Powerful Approach to Multiple Testing. *Journal of the Royal Statistical Society. Series B (Methodological)*, *57*(1), 289–300.

Bernstein, D. P., Stein, J. A., Newcomb, M. D., Walker, E., Pogge, D., Ahluvalia, T., Stokes, J., Handelsman, L., Medrano, M., Desmond, D., & Zule, W. (2003). Development and validation of a brief screening version of the Childhood Trauma Questionnaire. *Child Abuse & Neglect*, *27*(2), 169–190. https://doi.org/10.1016/S0145-2134(02)00541-0

Bethlehem, R. A. I., Seidlitz, J., White, S. R., Vogel, J. W., Anderson, K. M., Adamson, C., Adler, S., Alexopoulos, G. S., Anagnostou, E., Areces-Gonzalez, A., Astle, D. E., Auyeung, B., Ayub, M., Bae, J., Ball, G., Baron-Cohen, S., Beare, R., Bedford, S. A., Benegal, V., … Alexander-Bloch, A. F. (2022). Brain charts for the human lifespan. *Nature*, *604*(7906), 525–533. https://doi.org/10.1038/s41586-022-04554-y

Busso, D. S., McLaughlin, K. A., Brueck, S., Peverill, M., Gold, A. L., & Sheridan, M. A. (2017). Child Abuse, Neural Structure, and Adolescent Psychopathology: A Longitudinal Study. *Journal of the American Academy of Child & Adolescent Psychiatry*, *56*(4), 321-328.e1. https://doi.org/10.1016/j.jaac.2017.01.013

Holz, N. E., Zabihi, M., Kia, S. M., Monninger, M., Aggensteiner, P.-M., Siehl, S., Floris, D. L., Bokde, A. L. W., Desrivières, S., Flor, H., Grigis, A., Garavan, H., Gowland, P., Heinz, A., Brühl, R., Martinot, J.-L., Martinot, M.-L. P., Orfanos, D. P., Paus, T., … Marquand, A. F. (2023). A stable and replicable neural signature of lifespan adversity in the adult brain. *Nature Neuroscience*, *26*(9), 1603–1612. https://doi.org/10.1038/s41593-023-01410-8

Joseph, J., Buss, C., Knop, A., de Punder, K., Winter, S. M., Spors, B., Binder, E., Haynes, J.-D., & Heim, C. (2023). Greater maltreatment severity is associated with smaller brain volume with implication for intellectual ability in young children. *Neurobiology of Stress*, *27*, 100576. https://doi.org/10.1016/j.ynstr.2023.100576

Luo, Q., Zhang, L., Huang, C.-C., Zheng, Y., Kanen, J. W., Zhao, Q., Yao, Y., Quinlan, E. B., Jia, T., Banaschewski, T., Bokde, A. L. W., Bromberg, U., Büchel, C., Flor, H., Frouin, V., Garavan, H., Gowland, P., Heinz, A., Ittermann, B., … IMAGEN consortium. (2020). Association between childhood trauma and risk for obesity: A putative neurocognitive developmental pathway. *BMC Medicine*, *18*(1), 278. https://doi.org/10.1186/s12916-020-01743-2

Mackes, N. K., Golm, D., Sarkar, S., Kumsta, R., Rutter, M., Fairchild, G., Mehta, M. A., Sonuga-Barke, E. J. S., & on behalf of the ERA Young Adult Follow-up team. (2020). Early childhood deprivation is associated with alterations in adult brain structure despite subsequent environmental enrichment. *Proceedings of the National Academy of Sciences*, *117*(1), 641–649. https://doi.org/10.1073/pnas.1911264116

Malave, L., van Dijk, M. T., & Anacker, C. (2022). Early life adversity shapes neural circuit function during sensitive postnatal developmental periods. *Translational Psychiatry*, *12*(1), 1–14. https://doi.org/10.1038/s41398-022-02092-9

Marquand, A. F., Rezek, I., Buitelaar, J., & Beckmann, C. F. (2016). Understanding Heterogeneity in Clinical Cohorts Using Normative Models: Beyond Case-Control Studies. *Biological Psychiatry, Obsessive-Compulsive Disorder*, *80*(7), 552–561. https://doi.org/10.1016/j.biopsych.2015.12.023

Nelson, C. A., Sullivan, E. F., & Valdes, V. (2025). Early adversity alters brain architecture and increases susceptibility to mental health disorders. *Nature Reviews Neuroscience*, *26*(10), 642–656. https://doi.org/10.1038/s41583-025-00948-9

Paquola, C., Bennett, M. R., Hatton, S. N., Hermens, D. F., Groote, I., & Lagopoulos, J. (2017). Hippocampal development in youth with a history of childhood maltreatment. *Journal of Psychiatric Research, 91*, 149–155. https://doi.org/10.1016/j.jpsychires.2017.03.019

R Core Team. (2024, June). *R: A Language and Environment for Statistical Computing*. R Foundation for Statistical Computing. https://cran.r-project.org/

Teicher, M. H., Samson, J. A., Anderson, C. M., & Ohashi, K. (2016). The effects of childhood maltreatment on brain structure, function and connectivity. *Nature Reviews Neuroscience*, *17*(10), 652–666. https://doi.org/10.1038/nrn.2016.111

Vaidya, N., Marquand, A. F., Nees, F., Siehl, S., & Schumann, G. (2024). The impact of psychosocial adversity on brain and behaviour: An overview of existing knowledge and directions for future research. *Molecular Psychiatry*, *29*(10), 3245–3267. https://doi.org/10.1038/s41380-024-02556-y

Wang, H. R., Liu, Z.-Q., Pozzi, E., Hussain, A., Sigar, P., Abdallah, C., Alexander, N., Baker, J., Bauer, J., Blackford, J. U., Cisler, J., Connolly, C. G., Cotton, A., Daniels, J., Dannlowski, U., Densmore, M., deRoon-Cassini, T., Dima, D., Plessis, S. D., … Ho, T. C. (2026). Childhood Maltreatment and Deviations From Normative Brain Structure: A Mega-Analysis of 3711 Individuals From the ENIGMA Major Depressive Disorder and ENIGMA Posttraumatic Stress Disorder Working Groups. *Biological Psychiatry*, *0*(0). https://doi.org/10.1016/j.biopsych.2026.02.016

Zughayyar, I., Bauer, M., Güttler, C., de Almeida Marcelino, A. L., Kühne, F., Buss, C., Heim, C., Aigner, A., Tietze, A., & Dell’Orco, A. (2025). A FastSurfer Database for Age-Specific Brain Volumes in Healthy Children: A Tool for Quantifying Localized and Global Brain Volume Alterations in Pediatric Patients. *Brain and Behavior*, *15*(7), e70689. https://doi.org/10.1002/brb3.70689

Maltreatment Predicts Centiles of Individual Variation from Normative Brain Growth in Children

COMMSBIO-26-4414-A

Mohn et al.

Revised Manuscript

September 11, 2026

POINT-BY-POINT RESPONSE TO REVIEWERS

We sincerely thank the editors and reviewers for their careful evaluation of our revised manuscript and for their additional constructive comments. We greatly appreciate the opportunity to further strengthen our work.

In response to this second round of review, we have refined the interpretation and presentation of our findings and clarified the scope of the conclusions supported by our data. Below, we provide detailed point-by-point responses and indicate changes made in the manuscript.

Responses to Reviewer 1 (R1)

We sincerely thank R1 for the additional thoughtful and constructive comments. We greatly appreciate the suggestions, which helped us to further refine the manuscript and strengthen the framing and interpretation of our findings.

1. **Comment #1: My primary comment in the original review concerned the added value of normative centiles relative to conventional volumetric analyses. I appreciate that the authors directly addressed this question with additional sensitivity analyses. Importantly, however, these analyses suggest that the incremental predictive/explanatory value of centiles is relatively modest. For example, discrimination of maltreatment status was weak for both approaches (AUCs approximately 0.57–0.65), and centiles did not improve prediction over raw volumes. Similarly, associations with cognitive and behavioural measures were generally comparable across the two approaches, although there were some**

instances in which centiles appeared somewhat more sensitive. I don't view this as a weakness that requires further analysis. Rather, I think it provides an opportunity to more clearly define the contribution of the study. The normative framework provides an intuitive, population-referenced way of expressing individual variation, but the present findings do not demonstrate a substantial improvement in explanatory or predictive performance over conventional volumetric approaches. I would encourage the authors to acknowledge this more explicitly in the manuscript and frame the contribution accordingly.

We thank R1 for this helpful comment. We agree that our previous framing did not sufficiently state the specific contribution of the normative approach in the present study. Our aim was not to demonstrate that centiles provide superior predictive performance compared with raw volumes. Rather, we intended to bring the population-referenced perspective increasingly adopted by neuroscientists and psychiatrists studying neurodevelopmental and psychiatric disorders to the study of childhood maltreatment. Conventional case-control studies establish whether groups differ on average but provide limited information about where individuals lie within population variation, i.e., what would typically be expected for their age and sex and how far they deviate from it, or about the heterogeneity underlying group-level differences. Normative modelling addresses these complementary questions. The additional sensitivity analyses conducted in response to the previous review are consistent with this distinction and provided no evidence that centiles outperform conventional volumetric measures.

In response to the present comment, we have substantially revised and reorganized the Discussion to make this rationale explicit. Drawing on recent normative modelling work in ADHD and autism, we now emphasize that the value of brain charts in the present study lies not only in bringing a population reference to the study of childhood maltreatment, but also in revealing the substantial individual heterogeneity underlying the average maltreatment-related group difference. We found that although exposed children had lower brain centiles on average, children spanned a broad range of the normative distribution and substantially overlapped with non-exposed children. Our perspective thus also motivates an important next question: which characteristics of maltreatment exposure and which individual or contextual factors contribute to this heterogeneity? (Discussion, p. 8):

"Normative brain charts complement conventional case-control comparisons by charting each child's brain volume against an age- and sex-specific reference distribution. Their value in the present study lies in expressing individual differences on an intuitive, population-referenced scale and helping to characterize heterogeneity that may be obscured by group averages. For example, normative modelling in ADHD showed that group-level differences were only weakly reflected at the individual level, challenging the notion of the "average patient" (Wolfers et al., 2020) and similar work in autism suggests that group-level differences can be driven by subgroups of individuals with extreme deviations (Bethlehem et al., 2020). In our study, despite lower mean centiles in the CM+ group, children in both groups spanned a broad and substantially overlapping range of the normative distribution."

2. **Comment #2: Relatedly, I would suggest somewhat tempering statements regarding the clinical implications of the centile measures. I agree that expressing brain volumes relative to age- and sex-normative distributions is intuitive and potentially clinically interpretable. However, the present study does not establish clinical utility or demonstrate that these measures can reliably identify individual children at risk. Indeed, predictive discrimination was relatively modest. I therefore suggest distinguishing more clearly between interpretability relative to a normative population and demonstrated clinical utility. The former is a strength of this approach; the latter will require further prospective validation.**

We thank the reviewer and agree that population-referenced interpretability should be distinguished from clinical utility. Although normative centiles provide an intuitive way of expressing an individual child's brain measures relative to age- and sex-specific population expectations, our findings do not establish that these measures can identify individual children with maltreatment exposure or predict developmental outcomes. Indeed, the broad overlap in centile distributions between the CM+ and CM– groups reinforces this distinction.

We have therefore tempered the relevant statements in the Discussion and now explicitly state that the present findings do not establish the clinical utility of brain centiles. We further note that establishing whether normative brain measures provide predictive value for individual developmental outcomes will require prospective validation in independent samples (Discussion, p. 8):

“[…] Thus, lower average brain centiles associated with maltreatment do not constitute a uniform neuroanatomical phenotype at the individual level, nor do our findings establish clinical utility of brain centiles for identifying individual children with maltreatment exposure. Establishing clinical utility will require prospective validation of their predictive performance for developmental outcomes in independent samples. The observed variation was not clearly explained by maltreatment severity, for which associations with brain centiles did not survive correction for multiple comparisons. Understanding why children with maltreatment exposure occupy markedly different positions within the normative distribution, and whether factors associated with vulnerability or resilience contribute to this variation, represents important questions for future longitudinal studies.

3. **Comment #3: I also encourage the authors to carefully review references to altered or deviant “developmental trajectories.” The normative centiles are derived from age-related developmental reference curves, but the present study itself is cross-sectional. Thus, the data demonstrate that children occupy different positions relative to age- and sex-normative distributions; they do not directly demonstrate altered within-child trajectories of brain development. The authors appropriately acknowledge the cross-sectional design as a limitation, and I think ensuring that this distinction is maintained consistently throughout the manuscript would strengthen the interpretation.**

We thank R1 for this important comment and fully agree that our cross-sectional data cannot establish altered within-child trajectories of brain development. We have carefully reviewed the manuscript throughout and revised statements that could be interpreted as implying altered or deviant developmental trajectories, or causal effects of maltreatment on brain development. We now consistently describe our findings as differences in brain structure associated with maltreatment and as differences in children’s positions relative to age- and sex-normative distributions. For example, instead of *“we report substantial differences in age- and sex-related brain growth between children with and without confirmed maltreatment”* (Discussion, p. 7), which could suggest we assessed children’s brain development longitudinally, we now refer to *“differences in brain structure associated with childhood maltreatment”* (Discussion, p. 7) and report that children with maltreatment exposure “*showed lower brain centiles”* (Discussion, p. 7). References to developmental trajectories are restricted to prior longitudinal evidence or to the normative developmental

models underlying the brain charts, where appropriate. We have also ensured that the Discussion and Conclusions clearly distinguish the developmental reference provided by the brain charts from the longitudinal evidence that would be required to establish within-child developmental change. We thank the reviewer for highlighting this distinction, which has helped us refine the interpretation of our findings.

4. **Comment #4: Finally, the statement that "severity of maltreatment was associated with lower brain centiles" appears somewhat stronger than the corrected analyses support. Following FDR correction, the continuous severity associations with cGM, WM, and sGM centiles were not statistically significant (pFDR = .066, .066, and .172, respectively), although children with higher severity ratings showed lower centiles relative to the population norm. I suggest revising the abstract and related statements so that they accurately reflect this more nuanced result.**

We thank R1 for noting this ambiguity and agree that our wording should reflect the corrected analyses clearly. Although the continuous associations with cortical grey and white matter centiles were nominally significant, none survived correction for multiple comparisons. We have therefore sharpened the wording in the Results to distinguish the findings from these continuous severity analyses from the finding that children with higher severity ratings showed lower brain centiles relative to the population norm (Results, p. 5):

"To characterize the relationship between maltreatment severity and brain centiles, we defined maltreatment severity as the highest maltreatment rating across all maltreatment types a child had experienced. Maltreatment severity, modelled as a continuous predictor, was not significantly associated with cGM ($\beta = -0.06$, 95% CI [−0.11, −0.00], $t(112.63) = -2.04$, $p = 0.044$, $p_{FDR} = 0.066$), WM ($\beta = -0.06$, 95% CI [−0.11, −0.01], $t(115.00) = -2.32$, $p = 0.022$, $p_{FDR} = 0.066$), or sGM ($\beta = -0.04$, 95% CI [−0.08, 0.02], $t(114.99) = -1.38$, $p = 0.172$, $p_{FDR} = 0.172$) after controlling for multiple comparisons, but children who experienced maltreatment with a severity rating of 3 or higher had on average significantly lower brain centiles than the population norm (Figure 2)."

Most importantly, we revised the Abstract to reflect the main findings as follows:

"In our sample, children with maltreatment exposure exhibit lower mean brain centiles across all tissues than children with no exposure. Associations of greater maltreatment severity with lower brain centiles do not survive correction for multiple comparisons. Lower brain centiles are associated with lower intellectual ability and more behavioural problems."

Responses to Reviewer 2 (R2)

We thank R2 for her positive evaluation of our study. We appreciate the many thoughtful and detailed comments that have been a major help for us in the previous revision and her renewed enthusiasm for our results.

1. **Comment #1: The response to my comments on the last paragraph of page 9 are a strong argumentation for the novelty of the dataset. It is worth folding this into the introduction or discussion to highlight that.**

We thank R2 for this helpful suggestion. We agree, the detailed clinical characterization of maltreatment exposure and our child-adapted state-of-the-art neuroimaging protocol are important strengths of our dataset. We have incorporated this point explicitly as a strength into the main text, both in the Introduction and Discussion.

Introduction (p. 4):

"We recruited children with documented maltreatment exposure verified through structured clinical interviews (Cicchetti et al., 2003) and children without significant early adversity. This clinically well-characterized cohort addresses an important limitation of the existing literature, in which maltreatment has frequently been assessed retrospectively in adults, while neuroimaging studies of children with clinically verified exposure remain scarce (Teicher et al., 2016)."

Discussion (p. 10):

"A particular strength of this study is the combination of clinically assessed maltreatment exposure in a large sample of children with child-adapted state-of-the-art MRI acquisition and processing, thus addressing important limitations of a literature that has largely relied on retrospective assessment of childhood maltreatment in adults."

Building on the detailed contextualization provided in the previous versions of our manuscript, we have further expanded the Introduction to more clearly outline the research gap addressed by our study (Introduction, p. 3):

"More recently, normative modelling has extended this work by demonstrating that broad categories of adverse experiences are associated with deviations from typical developmental trajectories in adulthood (Holz et al., 2023), whereas a recent large-scale study found no significant associations between maltreatment and normative brain measures in its adolescent age group (Wang et al., 2026). However, whether maltreatment-related deviations from developmental benchmarks already detectable during childhood—a period of rapid brain growth and heightened neuroplasticity—remains unclear and requires neuroimaging studies in children with verified maltreatment exposure."

Finally, we now discuss differences to the study by Wang and colleagues that may explain the discrepant findings between our study and theirs, highlighting the importance for clinical assessments of maltreatment like in our study:

"Notably, a recent large-scale normative modelling study found no significant associations with brain structure in its child and adolescent age group (Wang et al., 2026). Differences in the assessment of maltreatment may contribute to these discrepant findings, as maltreatment in that study was assessed using questionnaire self-report, whereas the present study used detailed clinical assessment of exposure in children recruited through child welfare and protection services.

2. **Comment #2: This is just a minor aesthetic comment: experiment with increasing the font size within the matrix entries for Figure 3.**

We thank R2 for this suggestion. A higher-quality version of Figure 3 is provided with the submission as a separate figure file. We also included scatterplots of the bivariate associations in the Supplementary Materials (Supplementary Figure S3). In addition, associations between brain, cognitive and behavioural measures are now summarized in a separate table in the main manuscript (Table 3) to improve the clarity and readability of the Results section.

We are grateful to the Editor and both reviewers for their continued consideration of our work and for the constructive comments during this second round of review. We believe

that the resulting revisions have further strengthened the clarity and interpretation of our manuscript, and we hope that we have satisfactorily addressed all remaining concerns.

References

Bethlehem, R. A. I., Seidlitz, J., Romero-Garcia, R., Trakoshis, S., Dumas, G., & Lombardo, M. V. (2020). A normative modelling approach reveals age-atypical cortical thickness in a subgroup of males with autism spectrum disorder. *Communications Biology*, *3*(1), 486. https://doi.org/10.1038/s42003-020-01212-9

Cicchetti, D., Toth, S. L., & Manly, J. T. (2003). Maternal maltreatment classification interview. *Unpublished Manuscript, Mt. Hope Family Center, Rochester, NY*.

Holz, N. E., Zabihi, M., Kia, S. M., Monninger, M., Aggensteiner, P.-M., Siehl, S., Floris, D. L., Bokde, A. L. W., Desrivières, S., Flor, H., Grigis, A., Garavan, H., Gowland, P., Heinz, A., Brühl, R., Martinot, J.-L., Martinot, M.-L. P., Orfanos, D. P., Paus, T., … Marquand, A. F. (2023). A stable and replicable neural signature of lifespan adversity in the adult brain. *Nature Neuroscience*, *26*(9), 1603–1612. https://doi.org/10.1038/s41593-023-01410-8

Teicher, M. H., Samson, J. A., Anderson, C. M., & Ohashi, K. (2016). The effects of childhood maltreatment on brain structure, function and connectivity. *Nature Reviews Neuroscience*, *17*(10), 652–666. https://doi.org/10.1038/nrn.2016.111

Wang, H. R., Liu, Z.-Q., Pozzi, E., Hussain, A., Sigar, P., Abdallah, C., Alexander, N., Baker, J., Bauer, J., Blackford, J. U., Cisler, J., Connolly, C. G., Cotton, A., Daniels, J., Dannlowski, U., Densmore, M., deRoon-Cassini, T., Dima, D., Plessis, S. D., … Ho, T. C. (2026). Childhood Maltreatment and Deviations From Normative Brain Structure: A Mega-Analysis of 3711 Individuals From the ENIGMA Major Depressive Disorder and ENIGMA Posttraumatic Stress Disorder Working Groups. *Biological Psychiatry*, *0*(0). https://doi.org/10.1016/j.biopsych.2026.02.016

Wolfers, T., Beckmann, C. F., Hoogman, M., Buitelaar, J. K., Franke, B., & Marquand, A. F. (2020). Individual differences v. the average patient: Mapping the heterogeneity in

ADHD using normative models. *Psychological Medicine*, *50*(2), 314–323. https://doi.org/10.1017/S0033291719000084
