# Supplementary material for: Maltreatment predicts centiles of individual variation from normative brain growth in children
Source: Commun Biol. 2026 Oct 22;9:1465. doi: 10.1038/s42003-026-11143-6 (PMC13736999; doi:10.1038/s42003-026-11143-6)
Supplement: Supplementary file 2 [file 42003_2026_11143_MOESM2_ESM.pdf]

Supplementary Materials

Maltreatment predicts centiles of individual variation from normative brain growth in children

Johannes Julius Mohn, Martin Bauer, Ferdinand Hoffmann, Jerod Rasmussen, Anna Tietze, Sonja Entringer, Sibylle Winter, Claudia Buss & Christine Heim

Contains:

Supplementary Table S1-S4

Supplementary Figure S1-S3

Supplementary Table S1. Associations of maltreatment severity with brain centiles.

Imaging Phenotype	Estimate	95 % CI	*t*	df	*p*	Adjusted *p*
cGM	−0.06	−0.11, −0.00	−2.04	112.63	0.044*	0.066
WM	−0.06	−0.11, −0.01	−2.32	115.00	0.022*	0.066
sGM	−0.04	−0.09, 0.02	−1.38	115.00	0.172	0.172

Note. Estimates and 95% confidence intervals are beta coefficients for the Maltreatment Severity term from linear mixed-effects models adjusted for age and sex with a random intercept for family. *P* values were adjusted for multiple comparisons using the false discovery rate. Asterisks denote statistical significance: * $p < 0.05$, ** $p < 0.01$, *** $p < 0.001$.

Supplementary Table S2. Moderation of maltreatment–brain centiles associations by socioeconomic status.

Imaging Phenotype	Term	Estimate	95 % CI	*t*	df	*p*	Adjusted *p*
cGM	CM x SES	−0.09	−0.18, −0.00	−2.00	269.49	0.046*	0.138
WM	CM x SES	−0.02	−0.10, 0.06	−0.46	274.02	0.643	0.643
sGM	CM x SES	−0.04	−0.04, 0.03	−1.21	271.42	0.226	0.339

Note. Estimates and 95% confidence intervals are beta coefficients for the Maltreatment Group x SES term from linear mixed-effects models adjusted for age and sex with a random intercept for family. *P* values were adjusted for multiple comparisons using the false discovery rate. Asterisks denote statistical significance: * $p < 0.05$, ** $p < 0.01$, *** $p < 0.001$.

Supplementary Table S3. Moderation of associations among brain centiles, intellectual ability and behavioural problems by maltreatment status.

Phenotype	Term	Estimate	95% CI	*t*	df	*p*	Adjusted *p*
SONR	cGM x CM	−8.22	−20.81, 4.37	−1.29	282.42	0.200	0.570
WISC	cGM x CM	−12.74	−26.23, 0.76	−1.86	285.96	0.064	0.570
SONR	WM x CM	−5.37	−19.84, 9.09	−0.73	279.89	0.465	0.781
WISC	WM x CM	−11.01	−25.94, 3.92	−1.45	256.13	0.148	0.570
SONR	sGM x CM	−0.00	−15.43, 15.42	−0.00	278.37	1.000	1.000
WISC	sGM x CM	7.02	−8.84, 22.88	0.87	251.10	0.384	0.768
Externalizing	cGM x CM	2.07	−0.93, 5.07	1.36	275.00	0.175	0.570
Internalizing	cGM x CM	0.01	−2.57, 2.60	0.01	273.72	0.992	1.000
Externalizing	WM x CM	0.13	−3.44, 3.69	0.07	275.00	0.943	1.000
Internalizing	WM x CM	−0.81	−3.70, 2.07	−0.56	215.37	0.578	0.781
Externalizing	sGM x CM	1.05	−2.74, 4.84	0.55	275.00	0.586	0.781
Internalizing	sGM x CM	−1.81	−4.83, 1.21	−1.18	186.66	0.238	0.570

Note. Estimates and 95% confidence intervals are beta coefficients for the Brain Centile x Maltreatment Group term from linear mixed-effects models adjusted for age and sex with a random intercept for family. *P* values were adjusted for multiple comparisons using the false discovery rate. Asterisks denote statistical significance: * $p < 0.05$, ** $p < 0.01$, *** $p < 0.001$.

Supplementary Table S4. Distribution of siblings and twins in the neuroimaging sample.

	Total (n = 299)	CM− (n = 176)	CM+ (n = 123)
**Siblings**			
Only child	245 (81.9%)	143 (81.3%)	102 (82.9%)
Sibling	54 (18.1%)	33 (18.8%)	21 (17.1%)
… of which			
sibling pairs	14	10	4
sibling trios	4	3	1
sibling quartets	1	–	1
dizygotic twin pairs	3	–	3
monozygotic twin pairs	2	2	–
Non-related sample size	269	158	111

Note. Values are *n* (%) unless otherwise indicated. Percentages are calculated relative to the corresponding group sample. Non-related sample size indicates the number of independent families represented after accounting for siblings and twins within the sample. CM+, childhood maltreatment exposure; CM−, non-exposed.

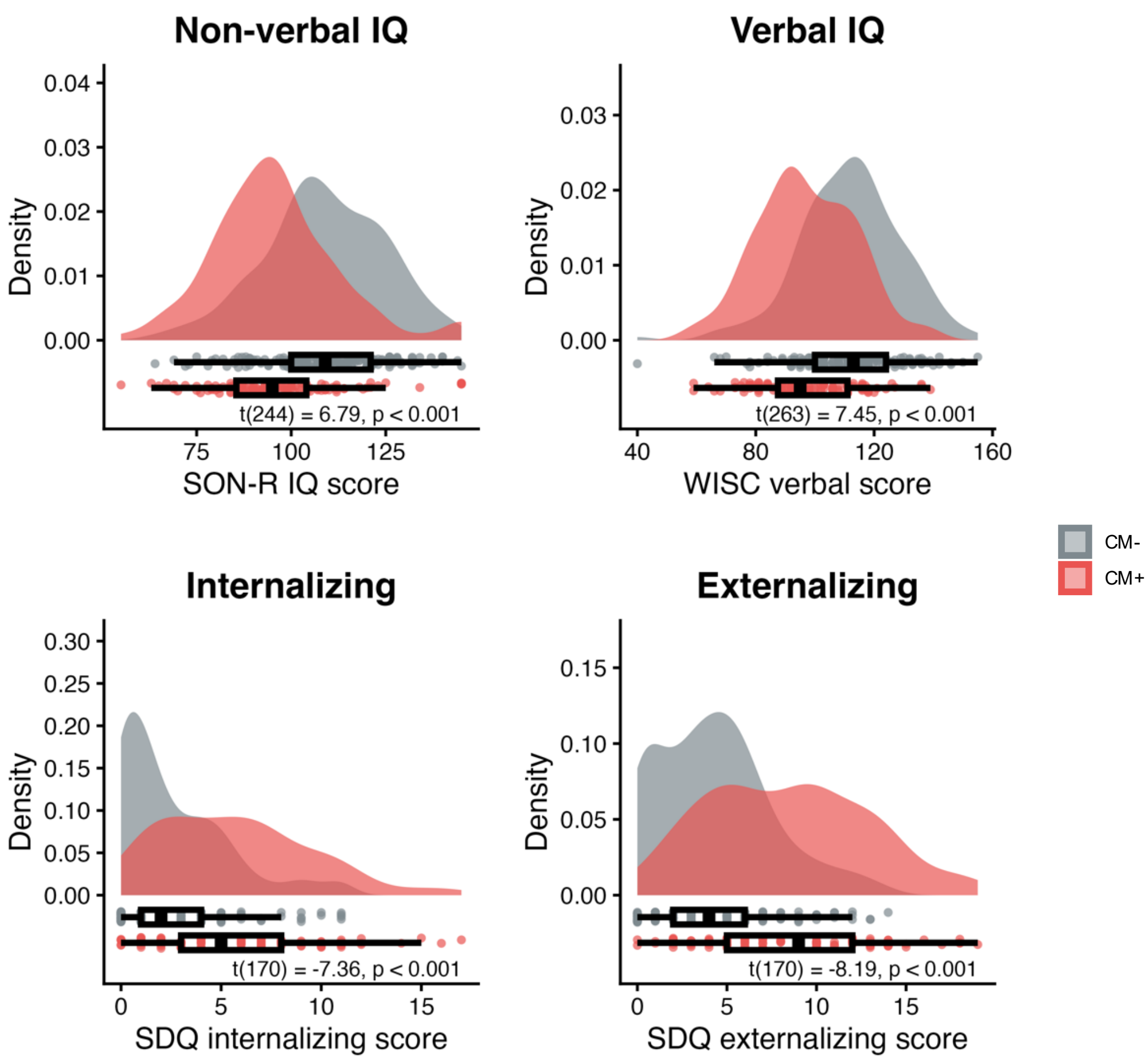

Supplementary Figure S1. Distributions and group differences in intellectual ability and behavioural problems. Distributions are shown for participants with (CM+, red) and without (CM−, grey) childhood maltreatment exposure. Unadjusted between-group differences were assessed using independent-samples *t*-tests (two-sided), with test statistics reported in each panel. SON-R, Snijders-Oomen Non-verbal Intelligence Test [1]; WISC, Verbal Comprehension Scale of the Wechsler Intelligence Scale for Children [2,3]; SDQ, Strengths and Difficulties Questionnaire [4].

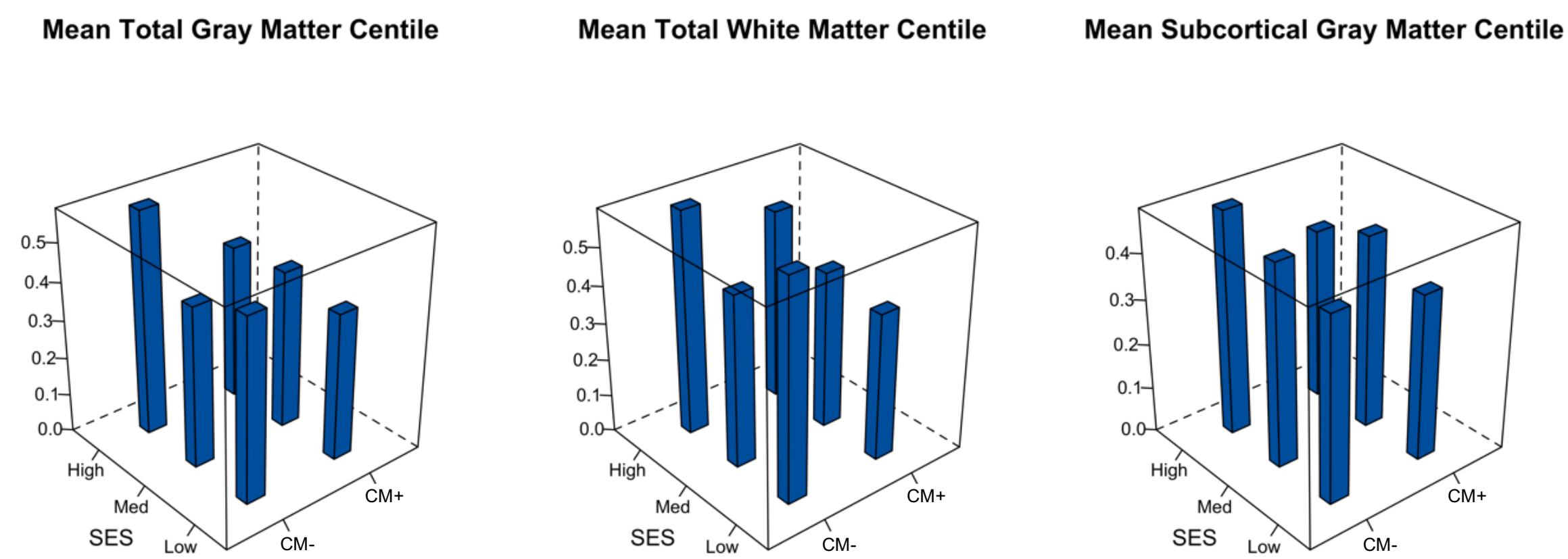

Supplementary Figure S2. Moderation of maltreatment–brain centile associations by socioeconomic status. Three-dimensional plots show brain centile scores according to childhood maltreatment exposure (CM+ vs. CM−) and socioeconomic status (SES). The *y*-axis represents brain centile scores, and bars show the mean centile score within each SES bin. Moderation was tested using linear mixed-effects models with SES modelled as a continuous variable, adjusting for age and sex and including a random intercept for family. No significant interactions between childhood maltreatment exposure and SES were observed for any brain centile measure after correction for multiple comparisons.

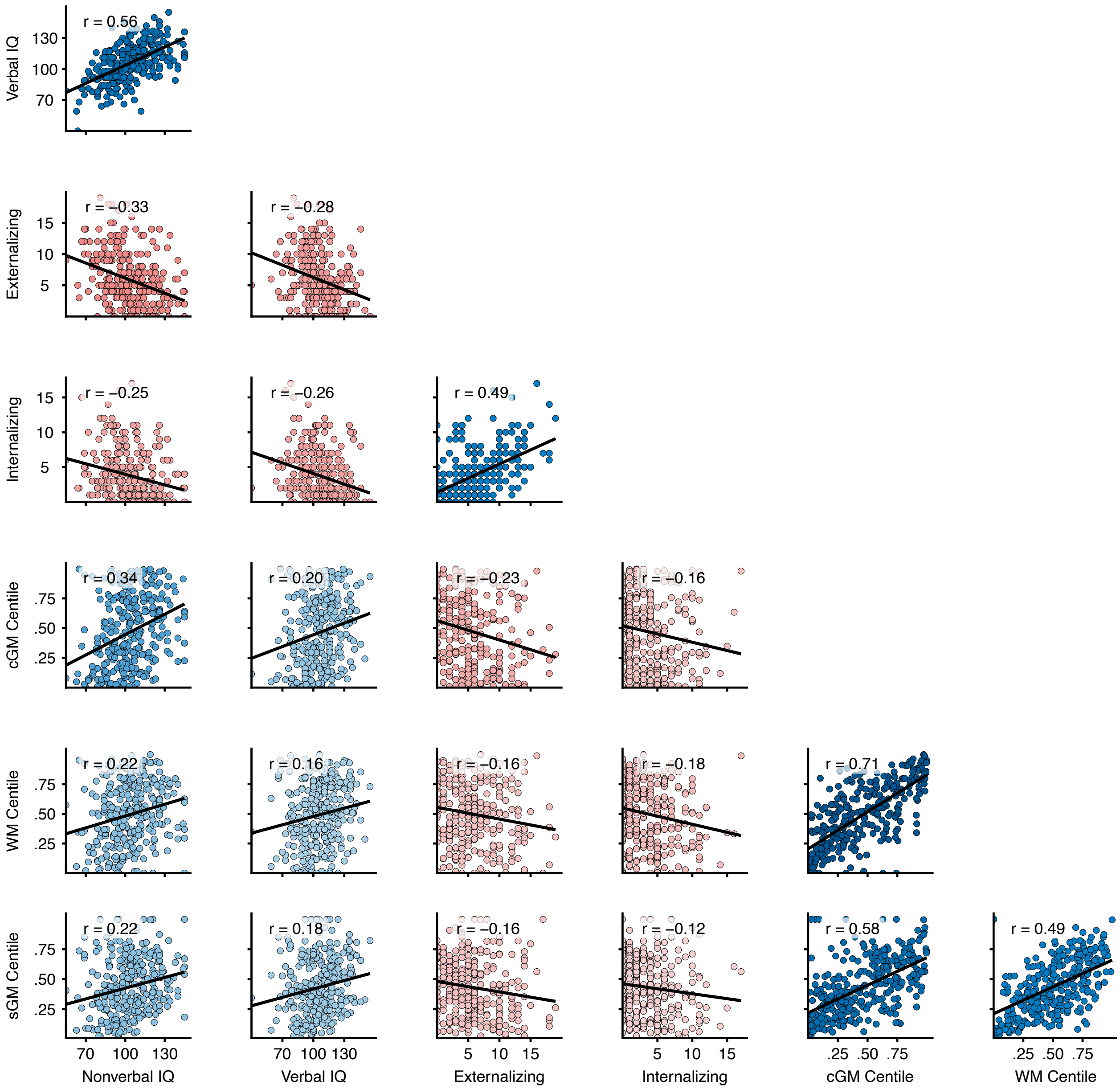

Supplementary Figure S3. Pairwise correlations between brain, cognitive and behavioural measures. Scatterplots show pairwise Pearson correlations between nonverbal IQ (SON-R[1]), verbal IQ (WISC-V[2,3]), externalizing and internalizing behaviours (SDQ[4]) and cortical grey matter (cGM), white matter (WM) and subcortical grey matter (sGM) brain centiles. Each point represents one participant and solid lines indicate linear fits. Black lines represent statistically significant correlations at $p < 0.05$, grey lines indicate non-significance. Pearson correlation coefficients (r) are shown within each panel. Colour indicates the direction and magnitude of the correlation coefficient, ranging from deep red for stronger negative correlations to deep blue for stronger positive correlations, with lighter colours indicating correlations closer to zero.

Supplementary References

1 Tellegen PJ, Laros JA. Snijders-Oomen Non-verbal Intelligence Test. Amsterdam: Hogrefe, 2011.

2 Wechsler D. Wechsler Preschool and Primary Scale of Intelligence—Fourth edition. San Antonio, TX: The Psychological Corporation, 2012.

3 Wechsler D. Wechsler Intelligence Scale for Children—Fifth Edition. Bloomington, MN: Pearson, 2014.

4 Goodman R. Psychometric properties of the Strengths and Difficulties Questionnaire. *J Am Acad Child Adolesc Psychiatry* 2001; **40**: 1337–45.
