# Supplementary material for: Maltreatment predicts centiles of individual variation from normative brain growth in children
Source: Commun Biol. 2026 Oct 22;9:1465. doi: 10.1038/s42003-026-11143-6 (PMC13736999; doi:10.1038/s42003-026-11143-6)
Supplement: Supplementary file 3 [file 42003_2026_11143_MOESM3_ESM.pdf]

Description of Additional Supplementary File

File name: Supplementary data

Description: Excel workbook containing the group-level source data underlying the main figures reported in the manuscript. Data are organized into separate worksheets corresponding to the relevant main figures. The individual-level data underlying this study are not publicly available due to participant privacy and data protection requirements and are available upon reasonable request to the corresponding author of the study.
