# Supplementary material for: Maltreatment predicts centiles of individual variation from normative brain growth in children
Source: Commun Biol. 2026 Oct 22;9:1465. doi: 10.1038/s42003-026-11143-6 (PMC13736999; doi:10.1038/s42003-026-11143-6)
Supplement: Supplementary file 4 [file 42003_2026_11143_MOESM5_ESM.pdf]

Please wait...

If this message is not eventually replaced by the proper contents of the document, your PDF viewer may not be able to display this type of document.

You can upgrade to the latest version of Adobe Reader for Windows®, Mac, or Linux® by visiting http://www.adobe.com/go/reader_download.

For more assistance with Adobe Reader visit http://www.adobe.com/go/acrreader.

Windows is either a registered trademark or a trademark of Microsoft Corporation in the United States and/or other countries. Mac is a trademark of Apple Inc., registered in the United States and other countries. Linux is the registered trademark of Linus Torvalds in the U.S. and other countries.
